# Deficits in Forelimb Reach Learning in a Mouse Model of Fragile X Syndrome

**DOI:** 10.1523/ENEURO.0126-25.2026

**Published:** 2026-04-14

**Authors:** Leanne F. Young, Ann Derham, Rui Zhu, Aparna Suvrathan

**Affiliations:** ^1^Centre for Research in Neuroscience, Brain Repair and Integrative Neuroscience Program, Research Institute of the McGill University Health Centre, Departments of Neurology and Neurosurgery; ^2^Pediatrics, McGill University, Montréal, Québec H3G 1A4, Canada; ^3^Integrated Program in Neuroscience, McGill University, Montréal, Québec H3G 1A4, Canada

**Keywords:** fragile X syndrome, learning, motor learning, mouse model

## Abstract

Fragile X syndrome is a leading cause of intellectual disability and autism spectrum disorder, for which therapies are limited. A mouse model of fragile X syndrome, the *Fmr1* knock-out (KO) mouse, has been particularly valuable for interrogating the molecular, cellular, and circuit mechanisms that underlie the neurological deficits seen in this syndrome. Key deficits in fragile X syndrome include impairments in social behaviors, cognition, and motor learning. Given the difficulties in extrapolating complex human behaviors to mouse models, motor behaviors are a particularly tractable form of learning to study in the mouse. We investigated a form of forelimb reach learning in both male and female *Fmr1* KO mice, quantifying different parameters of the task using both manual analysis and DeepLabCut-based tracking of reach trajectories. While *Fmr1* KO mice show impaired learning overall, our results showed that the presence or absence of a cue that signals reward alleviated some of the deficits. In addition to a single metric of success in learning, we determined the specific parameters of the motor behavior that were responsible for that success or failure. Our findings provide an essential framework for linking specific behavioral impairments in motor learning to the cellular and circuit mechanisms that support them.

## Significance Statement

Rodent models of neurodevelopmental disorders such as fragile X syndrome are key to understanding how cellular and circuit-level phenomena result in specific behavioral features. As a consequence, detailed and precise characterization of behavioral phenotypes in such mouse models is of critical importance. Here, we characterized to an unprecedented level of detail the deficits in a motor learning task in a mouse model of fragile X syndrome. In addition, we determined that the specific conditions of the behavioral task can provide an apparent alleviation of learning deficits overall. Our results provide a framework for determining the cellular and circuit deficits underlying these different behavioral features and identify how specific aspects of the learning deficit depend on the conditions of learning.

## Introduction

Fragile X syndrome (FXS) is the most common inherited form of intellectual disability and the leading monogenic cause of autism spectrum disorder (ASD), affecting approximately 1 in 5,000 males or 1 in 4,000–8,000 females, and causing 1–2% of all ASDs (Lévesque et al., 2009; Hunter et al., 2014; Aubertin, 2015; Hagerman et al., 2017). Despite its prevalence and the burden it imposes, effective therapies for FXS remain limited, largely due to our incomplete understanding of the disorder's neurobiological underpinnings (Belmonte and Bourgeron, 2006; Becker and Stoodley, 2013; Contractor et al., 2015; Hagerman et al., 2017). FXS arises from the loss of fragile X messenger ribonucleoprotein (FMRP, encoded by the *FMR1* gene), which is widely expressed in the brain and is involved in translational control of a wide array of mRNAs, many of which are critical for synaptic function and plasticity. Approximately 50–60% of males and 20% of females with FXS show symptoms of ASD, and intellectual deficits are widespread (Hagerman et al., 2017). The *Fmr1* knock-out (KO) mouse is a well-established model of FXS that replicates several of the signature features of FXS in humans: abnormal dendritic spine structure, altered synaptic plasticity, and behavioral deficits (The Dutch-Belgian Fragile X Consortium et al., 1994; Bear et al., 2004; Suvrathan et al., 2010; Santos et al., 2014; Hagerman et al., 2017; Schmitt et al., 2023).

Rodent models of FXS have permitted great strides in understanding the circuit, cellular, and molecular mechanisms underlying behavioral deficits. However, the ability to map neural functions to behavior requires detailed knowledge of the different aspects of behavior in the rodent model. In this context, motor function is a particularly appropriate and highly tractable behavior to investigate in rodents, allowing precise measurement and quantification of deficits.

Motor impairment is an important developmental characteristic in FXS and is also recognized more generally in ASD (Hampson and Blatt, 2015; Will et al., 2019; Wheeler et al., 2021). Moreover, comorbidity of ASD with FXS exacerbates the severity of motor phenotypes in children compared with those that have FXS alone (Will et al., 2019), and fine motor skills are associated with social communication skills (Taverna et al., 2021; Wheeler et al., 2021). Therefore, given the prevalence and importance of deficits in motor behavior, understanding the neural basis of these deficits is essential for developing strategies to alleviate them in FXS.

Despite this, detailed descriptions of motor deficits in rodent models of fragile X syndrome remain limited (Chen et al., 2022). Although there is evidence that pavlovian eyeblink conditioning is impaired (Koekkoek et al., 2005), our understanding of more complex forms of goal-directed motor learning remains incomplete. In turn, this lack of understanding limits our ability to link cellular/circuit deficits to specific aspects of motor function and motor learning. A key aim of therapeutic strategies in mouse models is to reverse the behavioral phenotype, highlighting the necessity for detailed, comprehensive characterization. In addition, animal and human behavior is complex and multifactorial, yet they are often reduced to a single simple score. In contrast, the ability to link cellular and circuit function to behavior necessitates a precise and detailed analysis of behavior in mouse models.

To address this knowledge gap, we investigated a form of goal-directed forelimb reach learning, in which several brain areas are involved, including the cerebellum (Becker and Person, 2019) and the motor cortex (Fu et al., 2012). In particular, the cerebellum has been strongly implicated in FXS, both in humans and in the mouse model (Mostofsky et al., 1998; Koekkoek et al., 2005; Huber, 2006), as has the motor cortex (Padmashri et al., 2013).

Skilled reaching is a powerful tool in sensorimotor neuroscience, offering high precision, rich kinematic resolution, and strong translational potential. The earliest systematic investigations were performed in humans, where directed movements of the hand were measured and described (Woodworth, 1899; Keele, 1968; Morasso, 1981), setting the stage for animal models. In nonhuman primates, foundational studies established that forelimb movements could be precisely quantified and were correlated with neural activity in the motor cortex (Evarts, 1968, 1973; Polit and Bizzi, 1978; Georgopoulos et al., 1986; Scott, 2003). More recent work has built a framework that complements principles deduced from human behavioral studies with cellular and systems-level mechanisms in animals (Graham et al., 2003; Churchland et al., 2006; Pizzimenti et al., 2007).

Parallel work in cats also provided key insights into propriospinal systems and their role in skilled motor control of the forelimb (Alstermark et al., 1981; Martin et al., 2005). Together, these studies showed that skilled forelimb reaching requires coordinated communication between brain areas and spinal cord circuits.

Skilled reach tasks were later adapted to rodents; the first detailed kinematic analysis of skilled forelimb reaching in rats showed that reaching is composed of a series of reproducible, segmental actions (Whishaw and Pellis, 1990). In rodents, forelimb reaching was characterized to provide a behavioral lexicon of movement components such as pronation, grasp, and supination, which can be used to assess motor system integrity (Whishaw and Pellis, 1990; Whishaw, 2004). This foundational framework has been extensively applied to study motor deficits following stroke and other neurological conditions in rats (Farr and Whishaw, 2002; Whishaw, 2003), including using deep learning methods to quantify motor deficits (Ryait et al., 2019). Systematic comparisons of skilled reaching in rats versus mice revealed conserved kinematic elements but also subtle species-specific differences (Whishaw and Coles, 1996). More recent work has shown that the task is sensitive to motor learning deficits and to perturbations in cortical circuits (Padmashri et al., 2013; Reiner and Dunaevsky, 2015; Suresh and Dunaevsky, 2023). Thus, given the available genetic tools, mice are a particularly suitable model.

Although the motor cortex was the earliest and most intensively studied region in the context of skilled reaching, it is now clear that it depends on multiple brain regions. The cerebellum, for instance, plays a key role in error signaling and reach adaptation (Becker and Person, 2019). Basal ganglia circuits and parietal regions have also been implicated, underscoring that skilled reaching depends on distributed circuits that integrate sensory feedback, planning, and motor execution (Tombaz et al., 2020; Dhawale et al., 2021).

The design of forelimb reach tasks has also been diversified to probe different scientific questions. Variants include lever-based tasks, button pressing, and robot manipulandum tasks, each of which allows for greater experimental control but reduces ethological naturalism (Vigaru et al., 2011; Fisher et al., 2014; Belsey et al., 2020; Wagner et al., 2021). The single-pellet retrieval version of the task preserves the full natural sequence of reaching components. This classical version involves a slit or narrow window cut into a plexiglass box where animals reach for single food pellets, an ethologically natural behavior suited to freely moving conditions (Whishaw and Pellis, 1990). This slit-based design has become the gold standard for assessing skilled motor function and has been extensively validated in a variety of studies, such as in assessing stroke recovery (Alaverdashvili and Whishaw, 2010; Nica et al., 2018; Salameh et al., 2020).

Performance metrics in the forelimb reaching task have been quite consistent across studies. Unlike binary operant tasks, skilled reaching is not constrained by chance success rates. Both rats and mice typically plateau at ∼40–60% success rates (Farr and Whishaw, 2002; Padmashri et al., 2013; Azim et al., 2014). These average success rates reflect the inherent difficulty of the task and provide a reliable benchmark across studies. Importantly, in addition to success rates, the single-pellet reaching task through a slit has proven particularly valuable for studying motor recovery following stroke, as well as deficits in genetically modified models (Alaverdashvili and Whishaw, 2010; Padmashri et al., 2013). Studies have shown that this task is sensitive to detecting deficits in reach trajectory, grasping, and retrieval components, allowing for quantitative success measures and fine-grained qualitative assessment of specific movement impairments (Azim et al., 2014; Becker and Person, 2019). This makes the task ideal for linking cellular and circuit mechanisms to specific aspects of motor dysfunction.

*Fmr1* KO mice exhibit clear deficits in forelimb reach learning, which were shown to be accompanied by impaired training-induced clustering of dendritic spines in motor cortex (Padmashri et al., 2013). More recently, impairments in the *Fmr1* KO mouse have also been shown to involve deficits in AMPAR insertion into dendritic spines during learning (Suresh and Dunaevsky, 2023). Together, these studies connect deficits in skilled reaching with underlying synaptic and circuit-level abnormalities, validating the task as a suitably sensitive assay for studying learning-related synaptic plasticity in *Fmr1* KO mice.

Here, we characterized deficits in forelimb reach learning in the *Fmr1* KO mouse, using both detailed manual analysis and automated markerless tracking (Mathis et al., 2018). We quantified a granular subdivision of categories of success and failure, highlighting that the overall reduction in success in *Fmr1* KOs could be broken down into differences in specific aspects of reach performance. Both manual and movement tracking analysis revealed that *Fmr1* KO mice are capable of improvement in some aspects of the task, even though learning is deficient relative to wild types. In addition, we described that the precise task parameters, specifically the presence of a cue, can change learning outcomes. Overall, this detailed characterization of forelimb reach learning in *Fmr1* KO provides an essential framework for understanding the neural basis of motor learning deficits in FXS.

## Materials and Methods

### Mice

All experiments were performed in accordance with the policies of the Canadian Council on Animal Care, using protocols approved by the Montreal General Hospital Facility Animal Care Committee. Mice of both sexes aged P55–P93 were used and were either wild type (WT), C57BL/6J (strain #000664, The Jackson Laboratory), or *Fmr1* KO (The Dutch-Belgian Fragile X Consortium et al., 1994; strain #003025, The Jackson Laboratory). Genotyping was performed following the protocol provided by The Jackson Laboratory and confirmed with automated genotyping services provided by Transnetyx. Mice were maintained on a 12 h inverted day–night cycle with *ad libitum* access to food and water except when food restricted for learning, as described below.

### Forelimb reaching task

**Figure 1.**
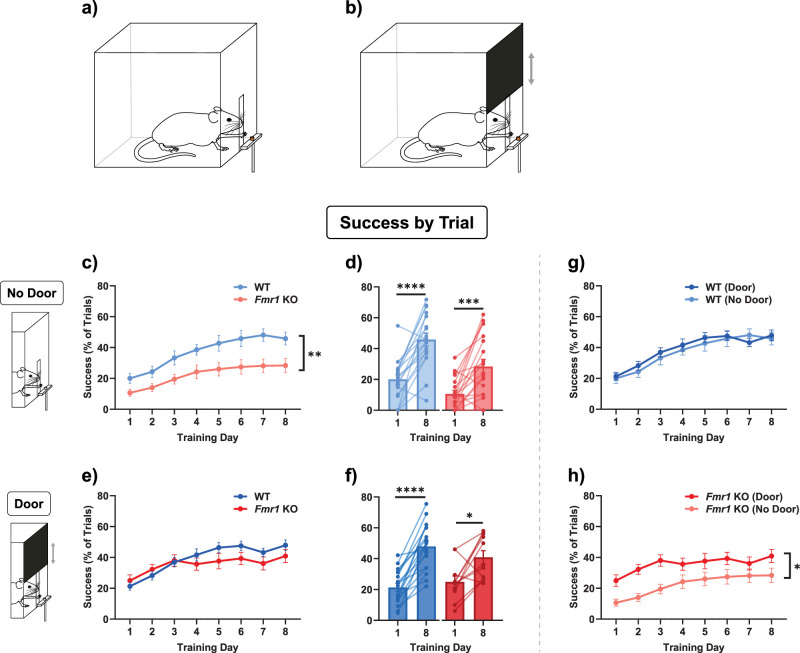
Forelimb reach learning was impaired in *Fmr1* KO mice. ***a***, Schematic of experimental setup illustrating the behavior box and slit through which the mouse had to reach to obtain a pellet reward. ***b***, Schematic of experimental setup modified to add an automated door that opened at trial start and closed at trial end. ***c***, Time course of forelimb reach learning. *Fmr1* KO mice have significantly lower success rates than those of wild-type (WT) mice in the No Door condition: time course of forelimb reach learning. ***e***, This deficit was alleviated in the Door condition. ***d, f***, Comparison of performances on Day 1 and Day 8 showed significant learning in both genotypes and in both conditions. ***g, h***, Same data as in ***c***–***f***; comparing Door and No Door conditions highlights that *Fmr1* KO mice performed significantly worse overall in the No Door condition. Time courses (***c, e, g, h***) were compared, based on the distribution of the data, either with a repeated-measures two-way ANOVA or with a mixed-effects model. Paired comparisons (***d, f***) were performed, based on the distribution of the data, either with a paired *t* test or with the Wilcoxon matched-pairs signed-rank test. The main effect of genotype is indicated in the line plots with asterisks. ****p < 0.0001, ***p < 0.001, *p < 0.05. Details of statistical tests are described in the Results section and summarized in Extended Data Figure 1-1.

All mice were handled for 5 min each day for 5 d before habituation to the behavior apparatus. Mice were food restricted to ∼85% of their original body weight starting 3 d prior to the first day of the experiment. The learning paradigm had three stages: “habituation” to introduce the mouse to the chocolate pellet food reward (Bio-Serv Dustless Precision Pellets, 20 mg, chocolate flavor) and learning environment, “shaping” to teach the mouse to use its paw to retrieve the food reward, and “training” which was the actual learning phase of the task. On the first day during habituation, each mouse was placed into a custom-built clear plexiglass box (“behavior box”) with a vertical 5 cm × 9 cm opening (“slit”) in the front wall (Fig. 1*a*,*b*) for 20 min. Food pellets were scattered in the box. Starting on the second day, mice had between 1 and 4 d of “shaping” to meet the requirements needed to pass onto the training stage. Each day of shaping was 20 min in duration. Shaping involved placing a food pellet on a platform outside but close to the slit, within reach of the mouse's tongue. Once the pellet was consumed, a new one was replenished, and the distance of the pellet to the slit was incrementally increased (to a maximum of 9 mm). This taught the mouse that the pellet was rewarding and introduced them to the idea of not using just the tongue to retrieve it. Then, one pellet was placed 1 cm away from the slit, and the mouse had to make a single reach attempt toward it. The criteria to pass shaping were a minimum of two pellets eaten from outside the slit, a maximum of three successful pellet retrievals at any distance, and a maximum of 10 reach attempts overall. All mice had to pass shaping before they could proceed to the training phase.

On the first day of training, all mice received a “reminder trial” where the pellet was placed into a 4-mm-wide indentation (“divot”) on a platform 1 cm away from the slit outside the behavior box. This trial was only given on the first day of training and did not count toward the trials used to analyze learning. Once the mouse made reach attempts that made contact with the pellet, it was removed, and the training day began. Mice underwent 8 consecutive days of training, and each training day consisted of 50 trials, where each trial was defined by a timed window where the mouse had access to the pellet before it was removed. Mice had 13 s of access to the pellet and 10 s of wait time between trials.

### Forelimb reaching task with an automated door

For some cohorts of mice, an automated door was attached to the behavior box to create consistent, tightly controlled camera recordings that could be synchronized with the trial structure. The door opened and closed at the same time intervals listed above to give mice the same amount of access time to the pellet as they did in the absence of the door. The protocol for the task remained the same, with the exception of including the activation of the automated door (14 s open, 8 s closed, including time to fully open and close) during shaping days to habituate the mice to the sound and sight of the door. The 13 s access period was defined as the window during which the pellet reward was physically accessible, timed from the moment the door cleared the height of the reward platform until it passed the same point during closing. Thus, there were two experimental conditions that differed only in the presence of the door; these are called the “No Door” and “Door” conditions (Fig. 1*a*,*b*).

### Manual reach scoring analysis

All behavioral experiments were recorded using a webcam at 60 frames per second (fps). Each reach was manually counted and classified according to a set of criteria, summarized in Table 1. A reach was defined as any reaching movement from when the paw dorsum passed outward through the slit and returned through the slit. A successful reach was defined as one continuous reaching and grasping motion where the pellet was successfully retrieved and brought to the mouse's mouth without letting the pellet touch the floor of the behavior box. A targeted reach was defined as any reach toward the pellet reward while it was on the platform. A vain reach was defined as a reach that was made even though there was no pellet reward present. Any targeted reach where the mouse was unable to bring the pellet back to its mouth was a failed reach: a complete miss if the mouse was unable to make any contact with the pellet and a contact miss if the mouse was able to touch the pellet but was unsuccessful in bringing it to the mouth. If a mouse did not perform any valid reaches during a trial, the trial was marked as “No Try.” A trial was considered successful if there was one successful reach attempt during the trial. The success rate calculated by successful trials did not include trials with no reach attempts.

**Table 1.** Reach classification summary table

Reach type	Description
First reach success	A successful reach made on the first attempt of the trial
Complete miss	A reach where the paw did not come into contact with the pellet
Contact miss	A reach where the paw came into contact with the pellet, but did not retrieve the pellet back past the slit. The pellet was not consumed during this trial
Vain	Pellet was not on the platform when the reach was made and was therefore not classified as a targeted reach. All other reach types require the pellet to be on the platform and are considered “targeted reaches”
Other	Any other reach that cannot be classified as one of the above categories

Summary table of the reach types and their descriptions used for behavioral scoring.

### Statistical methods

The time courses of learning were compared using a repeated-measures two-way ANOVA or, when values were missing (due to a lack of attempts by individual mice in some trials), a mixed-effects model. Paired comparisons between Day 1 and Day 8 performance were analyzed with either paired *t* tests or Wilcoxon matched-pairs signed-rank tests, depending on whether the data met assumptions of normality, which was assessed using a Shapiro–Wilk test. Unpaired comparisons were evaluated with Welch's *t* test since all data where this test was used were normally distributed.

All statistical analyses were conducted using GraphPad Prism Version 10.6.2 including *p*-values, *F*-statistics, and confidence intervals. The test statistic and *p*-value for both main and interaction effects are described in the Results section. All other statistical details, including effect size and confidence intervals, are provided in Extended Data Figure 1-1.

### 2D reach-tracking analysis with DeepLabCut

An opaque plexiglass door was added to the behavioral box and connected to a motor that was controlled by a Raspberry Pi running a custom Python script. The opening and closing of the door were synchronized to the activation and deactivation of the camera (OptiTrack Prime Color) used to record the mice. All recordings with this camera were made at an angled left side view at 240 fps, and every trial was recorded for each mouse over 8 d of learning the task. A subset of these videos was used to train a deep learning model to track the paws using DeepLabCut (Mathis et al., 2018).

#### DeepLabCut

All two-dimensional analysis was performed using DeepLabCut to track the paws and pellet. Over 2,500 frames were manually extracted from videos selected to maximize the diversity of mouse behaviors, and the left paw, right paw, nose, and pellet were manually labeled for each frame to train the network (ResNet-50). Every tracked element is labeled in every frame of an input video by DeepLabCut and is assigned a likelihood value; this score reflects the model's certainty in correctly identifying labels in a given frame. The “*p*-cutoff” is the threshold for filtering each tracked point, and data points falling below this threshold were excluded from further analysis. The *p*-cutoff was set to 0.85 to ensure only high-confidence data points were used for trajectory analyses. Since every frame is labeled, frames where the tracked elements (paws, nose, and/or pellet) were occluded when the mouse was, for example, facing away from the camera, were also assigned labels, but with a near-zero likelihood score. This filter removes those labels to ensure only data points where the paws are visible and confidently labeled are used for analysis. The 2,500+ labeled frames were split into a training set and a testing set, where 95% of the frames were used for training the model and 5% were used solely for testing model performance. Testing yielded high accuracy, achieving a 3.44 pixel (px) test error. Once the model had been trained on a subset of the videos, novel behavior videos were input into DeepLabCut, using the trained model to label and extract pixel coordinate points of all the tracked body parts and the reward pellet for further analysis.

#### Reach detection and extraction

Reach analysis and data filtering were conducted in Python. Tracking coordinates were filtered by set tracking bounds and the defined *p*-cutoff value described above. Individual reaches were then isolated from trial data using an algorithm. According to our operational definition, a reach is any movement in which the paw dorsum crosses the slit outward and subsequently retracts back through it. The tracking bounds were defined to encompass the area outside the behavior box where the paw extends beyond the slit to reach for the pellet. A reach was detected if it met a list of criteria: A reach must have at least six data points. If a data point and its subsequent five data points were within the tracking bounds, the vectors between each adjacent data point for 5 points succeeding the first were calculated $\left(\underset{P_{0}P_{1}}{\to },\;\underset{P_{1}P_{2}}{\to },\;\ldots ,\;\underset{P_{\left(n-1\right)}P_{n}}{\to }\right)$. If at least four out of five vectors were toward the direction of the pellet, the first data point of the sequence was marked as the start of a reaching movement. This process was then repeated for the opposite direction to determine the point at which the paw began retracting toward the slit, marked as the reach endpoint. This was continuously repeated, reversing the direction of vector comparisons until all tracked reaches in the trial had been exhausted. We analyzed trajectories without temporal filtering to avoid introducing smoothing artifacts or distortion. By excluding low-confidence detections using likelihood thresholds, the trajectories we obtained accurately followed paw position, as confirmed by manual checking.

### Code accessibility

The deep learning model used for movement tracking was trained and implemented using the DeepLabCut toolbox (Mathis et al., 2018). Reach extraction was performed as described above, using code written in Python (Python v3.9.5). All analyses were performed on a 64-bit workstation running Windows 10 Pro (Version 22H2), equipped with an Intel Core i9-10940X CPU (3.30 GHz), 64 GB of RAM and an NVIDIA GeForce RTX 3090 GPU (24 GB). The reach extraction code is accessible at https://github.com/Suvrathan-Lab/Young-Fmr1-Reach-Analysis.

## Results

### Success by trial

Goal-directed forelimb reach learning was tested in *Fmr1* KO mice versus WT mice (*N* = 67; see Materials and Methods for details). Mice were placed in a transparent box that had a slit on one wall, allowing access to a chocolate-flavored food pellet reward that was placed on a platform outside the slit (Fig. 1*a*,*b*). Mice had to learn to reach through the slit, grab the pellet reward, and retrieve it back through the slit to their mouths. The learning process took 8 d, with mice given 50 consecutive learning trials per day. Overall, *Fmr1* KO mice (*n* = 20; male *n* = 10, female *n* = 10) performed significantly worse than WT mice (*n* = 18; male *n* = 9, female *n* = 9), achieving a lower success rate at the task, as measured by the percentage of successful trials within the 50-trial experiment on each day, over 8 d (Fig. 1*c*; two-way repeated-measures ANOVA: main effect of genotype, *F*_(1,36)_ = 9.840, *p* = 0.0034; no interaction effect between genotype and training day, *F*_(4.153,149.5)_ = 1.266, *p* = 0.2853; effect size and confidence intervals are reported in Extended Data Fig. 1-1. For clarity, the effect of learning is separately quantified as the change from Day 1 to Day 8 in this and the following figures). However, both WT and *Fmr1* KO mice did learn to perform this task [Fig. 1*d*; Day 1 vs Day 8: paired *t* test (WT), *t*_(17)_ = 6.127, *p* < 0.0001; Wilcoxon test (*Fmr1* KO), *W* = 149.0, *p* = 0.0004]. This observation is consistent with previous experiments on a similar forelimb reach task (Padmashri et al., 2013).

10.1523/ENEURO.0126-25.2026.f1-1 Figure 1-1 Statistics table. Detailed statistical results table for all analyses in all figures, including test type, degrees of freedom, *F-*statistic (or other applicable statistic), *p*-values, effect sizes, and confidence intervals. Download Figure 1-1, TIF file.

To enable precise reach-tracking analysis, we optimized the task structure by introducing an automated door that defined the start and end of each trial (Fig. 1*b*; see Materials and Methods). Each trial began with the door opening, allowing the mouse access to the pellet for a fixed time interval before the door closed. This modification allowed for consistent, tightly controlled trial timing synchronized with video recordings. Surprisingly, the addition of this explicit cue marking trial onset improved the performance of *Fmr1* KO mice (*n* = 11; male *n* = 5, female *n* = 6), which no longer performed worse than WT mice (*n* = 18; male *n* = 9, female *n* = 9), although there was an interaction effect between genotype and training day (Fig. 1*e*; two-way repeated-measures ANOVA: no main effect of genotype, *F*_(1,27)_ = 0.7984, *p* = 0.3795; interaction effect of genotype and training day, *F*_(4.241,114.5)_ = 2.561, *p* = 0.0391). Both genotypes still improved at the task significantly (Fig. 1*f*; Day 1 vs Day 8: paired *t* test (WT), *t*_(17)_ = 8.306, *p* < 0.0001; Wilcoxon test (*Fmr1* KO), *W* = 52.0, *p* = 0.0186). These results indicate that the specific task parameters strongly influence behavioral performance and that the presence of the door cue alleviated part of the deficit observed in *Fmr1* KO mice (Fig. 1*g*,*h*; WT Door vs No Door, two-way repeated-measures ANOVA: no main effect of door, *F*_(1,34)_ = 7.015, *p* = 0.6758; no interaction effect between door and training day, *F*_(4.663,158.5)_ = 0.7717, *p* *=* 0.5632. *Fmr1* KO Door vs No Door, two-way repeated-measures ANOVA: main effect of door, *F*_(1,29)_ = 7.015, *p* = 0.0129; no interaction effect between door and training day, *F*_(3.668,106.4)_ = 0.9424, *p* = 0.4367).

### Success by reaches

**Figure 2.**
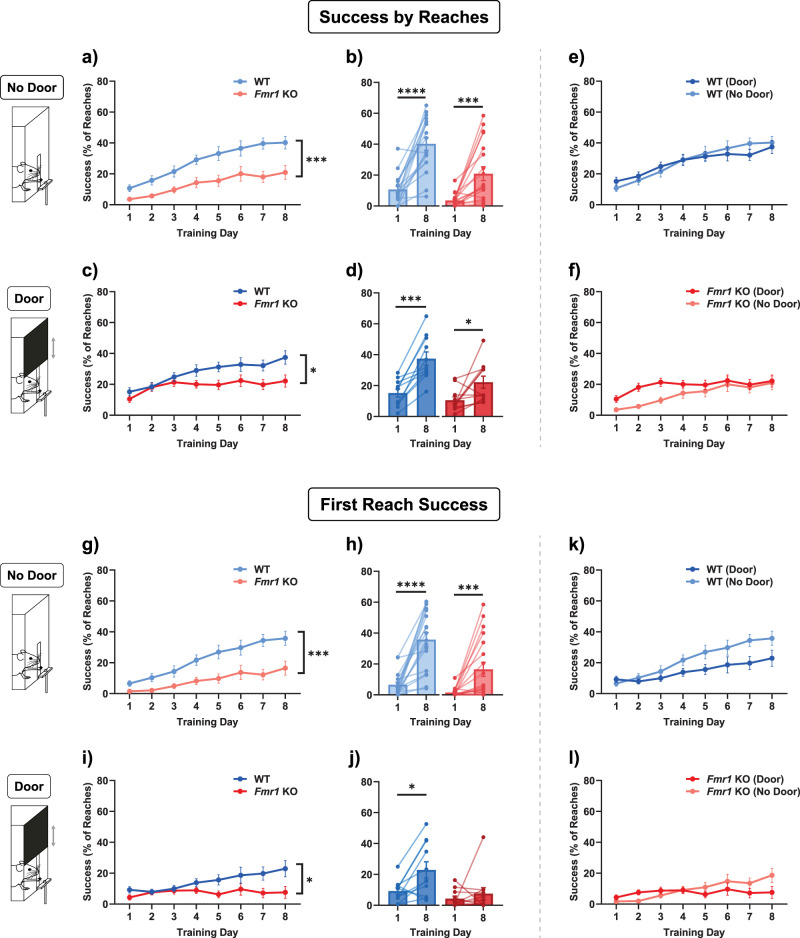
Forelimb reach learning was impaired in *Fmr1* KO mice when measured as a percentage of reaches. ***a, c***, *Fmr1* KO mice showed significantly impaired learning in both the No Door and the Door condition, with both a significant main effect of genotype and an interaction effect between genotype and training day. ***b, d***, Both genotypes showed significant learning from Day 1 to Day 8, under both conditions. ***e, f***, Same data as in ***a***–***c***, comparing Door versus No Door conditions. When success was measured as a percentage of reaches, there was no difference between the two door conditions. ***g, i***, The success of the first reach within a trial was also impaired in *Fmr1* KO mice relative to WT. ***h, j***, Both genotypes improved the rate of successful first reaches in the No Door condition, but only WT mice significantly improved in the Door condition. ***k, l***, Same data as in ***g–i***. There was no statistical difference between the Door and No Door conditions for both genotypes. Time courses (***a, c, e, f, g, i, k, l***) were compared with a two-way ANOVA with repeated-measures or with a mixed-effects model. Paired comparisons (***b, d, h, j***) were performed with a paired *t* test or Wilcoxon matched-pairs signed-rank test. The main effect of genotype is indicated in the line plots with asterisks. ****p < 0.0001, ***p = 0.001, *p < 0.05. Details of statistical tests are described in the Results section and summarized in Extended Data Figure 1-1. See also Extended Data Figures 2-1 and 2-2.

It has been well established that both individuals with fragile X syndrome and *Fmr1* KO mice have deficits in motor function and in motor learning. Therefore, we investigated this apparent alleviation of the deficit further. We reasoned that defining success by trial number neglects information about the actual number of reach attempts made by a given mouse during learning, which is a critical factor in motor learning. Thus, instead of defining success as the percentage of successful trials, we counted the number of reach attempts during each trial and measured the percentage of successful reaches out of the total targeted reach attempts (we defined targeted reaches as reaches that were made toward the food reward, in contrast to vain reaches, which were made when there was no food reward present at all). All the following results consider different parameters of learning in terms of the percentage of reaches. When analyzed in this manner, we observed that *Fmr1* KO mice do indeed have a deficit in learning (Fig. 2*a*–*d*) even with the door cue.

10.1523/ENEURO.0126-25.2026.f2-1 Figure 2-1 **Validation of behavioral scoring by scorers blind to genotype.** Reach analysis was performed by additional scorers who were blind to genotype, which replicated the results of Figure 2. a, b) *Fmr1* KO mice showed significantly impaired learning in both the No Door and the Door condition. Comparisons were done **using** a two-way ANOVA with repeated measures, or with a mixed-effects model. Main effect of genotype is indicated in the line plots by asterisks. *** *p* = 0.001, * *p* < 0.05. Details of statistical tests are described in the Results section and summarized in Figure 1-1: Statistics table. Detailed statistical results table for all analyses in all figures, including test type, **degrees of freedom, *F-*statistic (or other applicable statistic), *p*-values, effect sizes, and confidence intervals.** Download Figure 2-1, TIF file.

10.1523/ENEURO.0126-25.2026.f2-2 Figure 2-2 **Comparison of sex differences in WT and *Fmr1* KO mice. There was no difference in learning between sexes.** a-d) There was no significant difference between male and female mice in the time course of learning for both genotypes and for both Door and No Door conditions. Comparisons were done using a two-way ANOVA with repeated measures, or with a mixed-effects model. Details of statistical tests are described in the Results section and summarized in Extended Data Fig. 1-1. Download Figure 2-2, TIF file.

Reach success in both the Door and No Door experimental conditions was analyzed. In both cases (Fig. 2*a*,*c*), there was a significant impairment in learning in *Fmr1* KO mice [mixed-effects model (No Door, WT vs *Fmr1* KO): main effect of genotype, *F*_(1,36)_ = 14.22, *p* = 0.0006; interaction effect between genotype and training day, *F*_(2.981,106.5)_ = 2.718, *p* *=* 0.0486; two-way repeated-measures ANOVA (Door, WT vs *Fmr1* KO): main effect of genotype, *F*_(1,20)_ = 5.244, *p* = 0.033; interaction effect between genotype and training day, *F*_(2.836,56.72)_ = 2.838, *p* *=* 0.0488]. The Door and No Door conditions were not significantly different from each other [Fig. 2*e*,*f*; two-way repeated-measures ANOVA (WT Door vs No Door): no main effect of door, *F*_(1,27)_ = 0.02518, *p* = 0.8751; no interaction effect between door and training day, *F*_(3.677,99.27)_ = 1.362, *p* = 0.2548; mixed-effects model (*Fmr1* KO Door vs No Door): no main effect of door, *F*_(1,29)_ = 2.192, *p* = 0.1495; no interaction effect between door and training day, *F*_(2.176,62.47)_ = 1.915, *p* *=* 0.1524], and both WT and *Fmr1* KO mice learned from Day 1 to Day 8 [Fig. 2*b*,*d*; Day 1 vs Day 8: paired *t* test (WT, No Door), *t*_(17)_ = 7.014, *p* < 0.0001; Wilcoxon test (*Fmr1* KO, No Door), *W* = 145.0, *p* = 0.0001; paired *t* test (WT, Door), *t*_(10)_ = 6.039, *p* *=* 0.0001; paired *t* test (*Fmr1* KO, Door): *t*_(10)_ = 2.648, *p* = 0.0244].

Since the experiments and analyses were performed by the same person (L.F.Y.), they were not scored blind to condition. Therefore, two additional scorers (R.Z. and A.D.), who were blind to the genotype of the mouse, repeated this analysis and obtained the same results [Extended Data Fig. 2-1*a,b*; mixed-effects model (No Door, WT vs *Fmr1* KO): main effect of genotype, *F*_(1,36)_ = 14.22, *p* = 0.0006; interaction effect between genotype and training day, *F*_(2.981,106.5)_ = 2.718, *p* *=* 0.0486; two-way repeated-measures ANOVA (Door, WT vs *Fmr1* KO): main effect of genotype, *F*_(1,20)_ = 5.244, *p* = 0.033; interaction effect between genotype and training day, *F*_(2.836,56.72)_ = 2.838, *p* *=* 0.0488].

In patients with fragile X syndrome, due to the X-linked nature of the deficit, females often show milder phenotypes (Hagerman et al., 2017). However, in the mouse model, FMRP is absent in both sexes, and the degree of X inactivation does not play a role. Notwithstanding this difference between the mouse model and humans, sex differences have been described in the *Fmr1* KO mouse (Croom et al., 2024; but see also Baker et al., 2010). Therefore, in order to determine whether the sexes show differences in forelimb reach learning, we compared the behavior of males and females. We found no significant difference between the sexes, in either genotype, and in both the Door and No Door conditions (Extended Data Fig. 2-2*a–d*; see Extended Data Fig. 1-1 for statistical comparisons).

Success and failure during forelimb reach learning can arise from a variety of underlying factors. A binary classification of success versus failure conceals multiple possibilities: The paw did not reach the target at all; it reached the target but grasping or retrieval failed; the mouse required multiple consecutive reaches within a trial in order to refine its movement to obtain the target, as opposed to executing a successful reach, grasp, and retrieval on the first attempt of the trial; or the mouse may have made fewer attempts. To help disambiguate these scenarios, we performed a detailed manual analysis of each reaching movement.

### First reach success

First, we assessed whether a mouse had to make several reach attempts within a trial in order to successfully obtain the pellet reward. During these multiple sequential attempts, mice can improve the trajectory of their reach and get closer to the target. We observed that in both the No Door and Door conditions, *Fmr1* KO mice were significantly less able to retrieve the pellet reward on the first attempt of a trial, with an interaction effect in only the No Door condition [Fig. 2*g*,*i*; mixed-effects model (WT vs *Fmr1* KO, No Door): main effect of genotype, *F*_(1,36)_ = 14.37, *p* = 0.0006; interaction effect between genotype and training day, *F*_(2.713,96.90)_ = 3.589, *p* = 0.0197; two-way repeated-measures ANOVA (WT vs *Fmr1* KO, Door): main effect of genotype, *F*_(1,20)_ = 4.489, *p* = 0.0468; no interaction effect of genotype and training day, *F*_(2.443,48.85)_ = 2.888, *p* = 0.0551], although in the No Door condition, both WT and *Fmr1* KO mice improved after training [Fig. 2*h*; Wilcoxon test (WT): *W* = 171.0, *p* < 0.0001; Wilcoxon test (*Fmr1* KO): *W* *=* 126.0, *p* = 0.0003], and *Fmr1* KO mice did not in the Door condition [Fig. 2*j*; paired *t* test (WT): *t*_(10)_ = 3.115, *p* = 0.011; Wilcoxon test (*Fmr1* KO): *W* = 12.0, *p* = 0.6377]. The inability to make as many “first reach success” attempts highlights an important aspect of the learning deficit. As previously, there was no significant difference overall between the No Door and Door conditions, although there was an interaction effect between the door condition and training day in the *Fmr1* KO mice [Fig. 2*k*,*l*; two-way repeated-measures ANOVA (WT Door vs No Door): no main effect of door, *F*_(1,27)_ = 3.281, *p* = 0.0812; no interaction effect between door and training day, *F*_(3.535,95.44)_ = 2.423, *p* *=* 0.0607; mixed-effects model (*Fmr1* KO Door vs No Door): no main effect of door, *F*_(1,29)_ = 0.2949; *p* = 0.5913; interaction effect between the door and training day, *F*_(1.737,49.88)_ = 3.646, *p* = 0.0391].

### Failed reaches:

**Figure 3.**
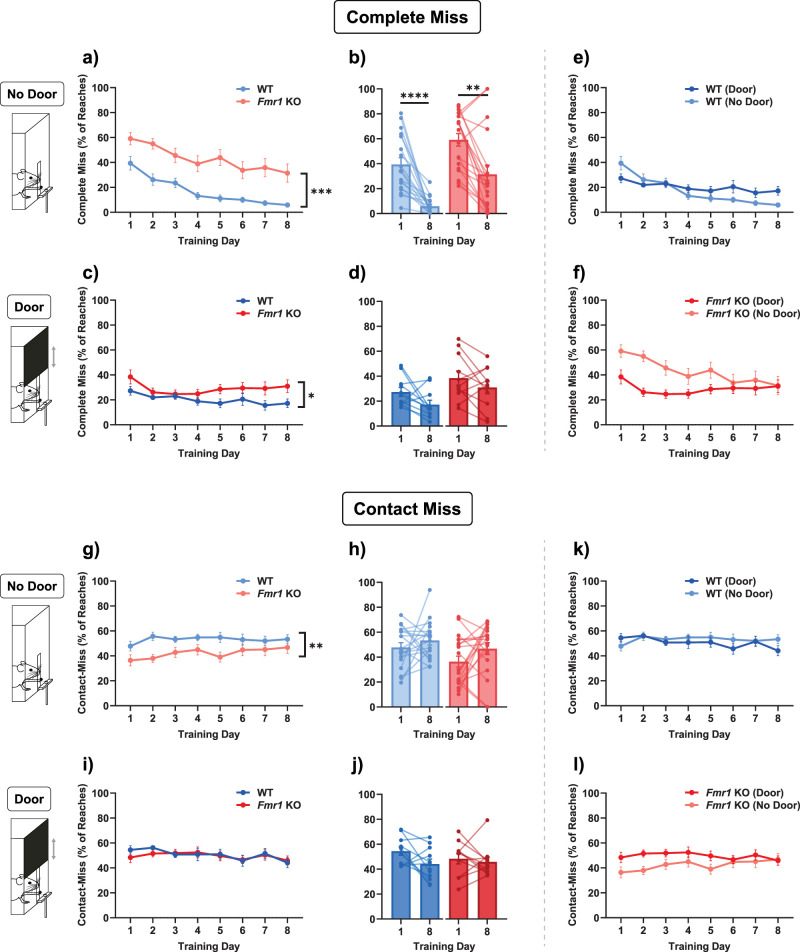
Learning was impaired in *Fmr1* KO mice when measured as a reduction in failures. ***a, c***, In both No Door and Door conditions, *Fmr1* KO mice were impaired in terms of the percentage of complete misses they made. ***b, d***, Both genotypes showed significant improvement of this metric only in the No Door condition, and not in the Door condition. ***e, f***, Same data as in ***a***–***c***; comparing Door versus No Door showed no significant genotype effect between the conditions; however, there was a significant interaction effect in both WT and *Fmr1* KO between genotype and training day. ***g, i***, *Fmr1* KO mice showed a lower number of contact misses only in the No Door condition. ***h, j***, In neither condition was there significant learning for either genotype. ***k, l***, Same data as ***g–j***; comparing Door versus No Door conditions showed no significant difference between the genotypes. Time courses (***a, c, e, f, g, i, k, l***) were compared with a two-way ANOVA with repeated-measures or with a mixed-effects model. Paired comparisons (***b, d, h, j***) were performed with a paired *t* test or Wilcoxon matched-pairs signed-rank test. The main effect of genotype is indicated in the line plots by asterisks. ****p < 0.0001, ***p = 0.001, **p < 0.01, *p < 0.05. Details of statistical tests are described in the Results section and summarized in Extended Data Figure 1-1.

Another feature of learning that is encompassed within the success rate metric is the precise way in which the reach was not successful. In particular, it was recorded as a failure both when the mouse completely failed to make contact with the pellet reward, defined as a “complete miss,” or when the mouse made a forelimb movement whose trajectory was appropriate enough to contact the pellet reward but then failed to grasp or retrieve it, which we defined as a “contact miss.” It is important to distinguish whether the learning deficit in *Fmr1* KO mice arises because of an aberrant reach trajectory or because of an inability to learn how to grasp and retrieve it. Therefore, we measured complete misses under both the No Door (Fig. 3*a*,*b*) and the Door (Fig. 3*c*,*d*) conditions and found that there was a significant impairment in *Fmr1* KO mice [Fig. 3*a*–*c*; mixed-effects model (WT vs *Fmr1* KO, No Door): main effect of genotype, *F*_(1,36)_ = 18.81, *p* = 0.0001; no interaction effect between genotype and training day, *F*_(2.9626,104.5)_ = 0.8845, *p* = 0.4496; Wilcoxon test (Day 1 vs Day 8, WT, No Door): *W* = −171.0, *p* < 0.0001; Wilcoxon test (Day 1 vs Day 8, *Fmr1* KO, No Door): *W* = −144, *p* = 0.0024; two-way repeated-measures ANOVA (WT vs *Fmr1* KO, Door): main effect of genotype, *F*_(1,20)_ = 4.759, *p* = 0.0413; no interaction effect between genotype and training day, *F*_(3.235,64.69)_ = 1.191, *p* = 0.3213]. While neither genotype showed significant learning with this metric in the Door condition, the deficit was more pronounced in the No Door condition [Fig. 3*d*; Wilcoxon test (Day 1 vs Day 8, WT, Door): *W* = −42.0, *p* = 0.0674; paired *t* test (Day 1 vs Day 8, *Fmr1* KO, Door): *t*_(10)_ = 1.413, *p* *=* 0.1879]. However, although there was no main effect of genotype on the progression of learning between the Door and No Door conditions, there was a significant interaction effect in both [Fig. 3*e*,*f*; two-way repeated-measures ANOVA (WT, Door vs No Door): no main effect of door, *F*_(1,27)_ = 1.140, *p* = 0.2951; interaction effect between door and training day, *F*_(2.825,76.28)_ = 3.321, *p* = 0.0265; mixed-effects model (*Fmr1* KO Door vs No Door): no main effect of door, *F*_(1,29)_ = 3.298, *p* = 0.0797; interaction effect between door and training day, *F*_(3.006,86.31)_ = 3.793, *p* = 0.0131]. We speculate that the presence of the door makes the task easier by providing some guidance to the mouse's paw. This is possible because mice attempt to reach the pellet reward as soon as the door begins opening, which means that the bottom of the door is still sliding upward when their paw reaches through the slit, creating a line of sight and physical barrier that is guided with the upward movement of the door. Therefore, they cannot make completely mistargeted reaches in the large open space above the reward while the door is in motion, which we often observed in the early stages of learning when there was no door.

In order to further categorize miss categories, we tested if there were differences in learning in terms of reduction of contact misses, where the mouse's paw successfully makes contact with the target but it either fails to grasp it or drops it before retrieval into the box. Again, there was a significant difference between WT and *Fmr1* KO mice, but this was only observed in the No Door condition (Fig. 3*g*; mixed-effects model: main effect of genotype, *F*_(1,36)_ = 7.555, *p* = 0.0093; no interaction effect, *F*_(2.959,105.7)_ = 0.8420, *p* = 0.4725) and not in the Door condition (Fig. 3*i*; two-way repeated-measures ANOVA: no main effect of genotype, *F*_(1,20)_ = 0.06402, *p* = 0.8028; no interaction effect, *F*_(3.850,76.99)_ = 0.6349, *p* = 0.6332). There was no significant difference between Day 1 and Day 8 in both conditions [Fig. 3*h*,*j*; paired *t* test (Day 1 vs Day 8, WT, No Door): *t*_(17)_ = 0.9896, *p* = 0.3362; paired *t* test (Day 1 vs Day 8, *Fmr1* KO, No Door): *t*_(18)_ = 1.579, *p* = 0.1317; paired *t* test (Day 1 vs Day 8, WT, Door): *t*_(10)_ = 2.163, *p* = 0.0558; Wilcoxon test (Day 1 vs Day 8, *Fmr1* KO, Door): *W* = −12.0, *p* = 0.6377]. The observation that *Fmr1* KO mice had fewer contact misses than WT mice might be a reflection of a higher number of complete miss reaches in the *Fmr1* KO. In neither condition was there a significant improvement over the time course of learning. This might suggest that, rather than an improved ability to grasp and retrieve the pellet once contact has been made, the ability to make an appropriate reach trajectory to the pellet reward is what mice are learning. In turn, this observation presented the need for further analysis of the movement, which led to our analysis of the reach trajectories (Fig. 6). Although the Door and No Door conditions showed markedly different results, a direct comparison between them over the course of learning did not attain significance [Fig. 3*k*,*l*; two-way repeated-measures ANOVA (WT, Door vs No Door): no main effect of door, *F*_(1,27)_ = 0.5750, *p* = 0.4548; no interaction effect between door and training day, *F*_(2.966,80.07)_ = 1.141, *p* = 0.3375; mixed-effects model (*Fmr1* KO, Door vs No Door): no main effect of door, *F*_(1,30)_ = 2.380, *p* = 0.1334; no interaction effect between door and training day, *F*_(3.242,93.09)_ = 1.219, *p* = 0.3077].

### Vain reaches

**Figure 4.**
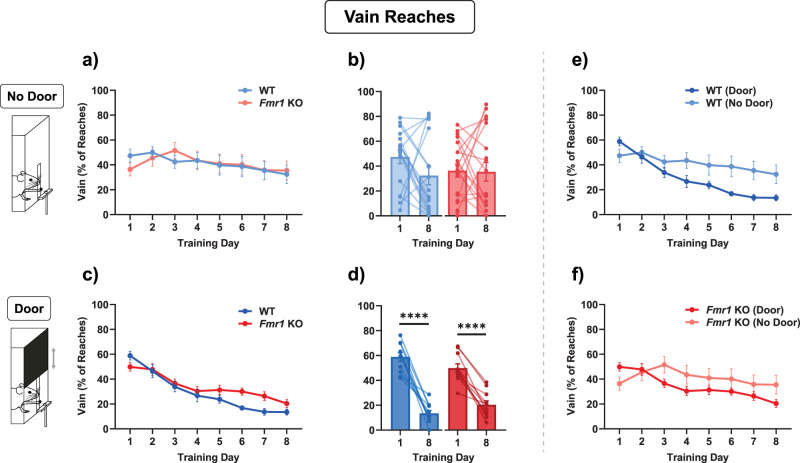
Vain reaches did not explain the lower success rates in *Fmr1* KO mice. ***a, c***, In both Door and No Door conditions, there was no difference in the time course of vain reaches as a percentage of total reaches; however, there was a significant interaction effect in the Door condition between genotype and training day. ***b, d***, Both genotypes learned only in the Door condition, and not in the No Door condition. ***e, f***, Same data as in ***a***–***c***, but comparing Door versus No Door conditions. There was no significant main effect of the door. Time courses (***a, c, e, f***) were compared with a two-way ANOVA with repeated-measures or with a mixed-effects model. Paired comparisons (***b, d***) were performed with a paired *t* test or Wilcoxon matched-pairs signed-rank test. ****p < 0.0001. Details of statistical tests are described in the Results section and summarized in Extended Data Figure 1-1. See also Extended Data Figure 4-1.

While observing mouse behavior during this task, we noticed that mice sometimes continued to reach even when there was no pellet reward present. Since the box was transparent and the reward pellet was placed directly in front of the slit, it should have been visible to the mice when there was no reward. Yet, they sometimes continued to make reach attempts regardless. We called such reaches “vain reaches.” Given that there is a tendency for repetitive behaviors in FXS (Reisinger et al., 2020), we wondered whether *Fmr1* KO mice showed either more vain reaches or less reduction in vain reaches over the time course of learning. Vain reaches were quantified as a percentage of total reaches, not just targeted reaches (Fig. 4*a*–*f*). There was no reduction in vain reaches in the No Door condition [Fig. 4*a*,*b*; mixed-effects model (WT vs *Fmr1* KO): no main effect of genotype, *F*_(1,36)_ = 0.004254, *p* = 0.9484; no interaction effect between genotype and training day, *F*_(2.299,82.11)_ = 0.9201, *p* = 0.414; Wilcoxon test (Day 1 vs Day 8, WT): *W* = −77.0, *p* = 0.0987; paired *t* test (Day 1 vs Day 8, *Fmr1* KO): *t*_(18)_ = 0.3167, *p* = 0.7551]. In the Door condition, there was an interaction effect between genotype and training day [Fig. 4*c*; two-way repeated-measures ANOVA (WT vs *Fmr1* KO): no main effect of genotype, *F*_(1,20)_ = 2.424, *p* = 0.1352; interaction effect between genotype and training day, *F*_(4.247,84.94)_ = 2.767, *p* = 0.0298]) Notably, in the Door condition, there was also a reduction of vain reaches, i.e., an improvement from Day 1 to Day 8, in both genotypes [Fig. 4*d*; paired *t* test (Day 1 vs Day 8, WT): *t*_(10)_ = 9.245, *p* < 0.0001; paired *t* test (Day 1 vs Day 8, *Fmr1* KO): *t*_(10)_ = 8.480, *p* < 0.0001]. Direct comparison of the Door and No Door conditions showed no significant difference in WT mice, although there was a significant interaction effect between the door condition and training days (Fig. 4*e*; two-way repeated-measures ANOVA: no main effect of door, *F*_(1,27)_ = 3.125, *p* = 0.0884; interaction effect between door and training day, *F*_(2.410,65.07)_ = 3.212, *p* = 0.038). Similarly, in the *Fmr1* KO mice, there was no overall main effect of the door, but there was an interaction effect [Fig. 4*f*; mixed-effects model: no main effect of door, *F*_(1,29)_ = 0.5769, *p* = 0.4537; interaction effect between door and training day, *F*_(3.075,88.31)_ = 3.106, *p* = 0.0295]. Therefore, this was yet another aspect of the learning task that is markedly different between the two experimental conditions we tested, with the presence of the door cue leading animals to make fewer vain reaches over the time course of learning.

10.1523/ENEURO.0126-25.2026.f4-1 Figure 4-1 **Comparison of “No Try” trials between WT and *Fmr1* KO mice in the No Door and Door conditions.** No Tries varied between the Door and No Door conditions. a, b) There was a reduction in No Tries in WT and *Fmr1* KO mice in the No Door condition, indicating an improvement in performance. c, d) The number of No Tries were comparably low on both Day 1 and Day 8 for WT and *Fmr1* KO mice in the Door condition. e, f) Same data as in a, c, comparing Door vs. No Door conditions between genotypes. Paired comparisons (b, d) were performed with a paired *t*-test or Wilcoxon matched pairs signed rank test. *** *p* = 0.001, * *p* < 0.05. Details of statistical tests are described in the Results section and summarized in Extended Data Fig. 1-1. Download Figure 4-1, TIF file.

### No try trials

In addition, we noticed that sometimes mice appeared to miss several trials just because they were distracted and were in another part of the box or only noticed that the reward pellet for the next trial was in position shortly before the end of the trial. We therefore defined another metric called “No Try” and counted the number of trials where the animals did not make reach attempts. Due to the low number of no try trials (zero values) across genotypes, repeated-measures two-way statistical comparisons were not possible (Extended Data Fig. 4-1*a,c*); however, there was a trend showing an increase in the number of missed trials in the No Door condition in *Fmr1* KO mice. It is possible that this was due to the inherent sound or visual cue of the door opening, which potentially acted as a signal to notify animals when to pay attention that a new trial was starting. Both WT and *Fmr1* KO mice showed a significant improvement in the reduction of missed trials in the No Door condition [Extended Data Fig. 4-1*b*; Wilcoxon test (WT): *W* = −124, *p* = 0.0004; Wilcoxon test (*Fmr1* KO): *W* = −109.0, *p* = 0.0409], while both genotypes already had comparably low trials with no tries from Day 1 to Day 8 in the Door condition [Extended Data Fig. 4-1*d*; Wilcoxon test (WT): *W* = −32.0, *p* = 0.0586; Wilcoxon test (*Fmr1* KO): *W* = −28, *p* = 0.0547] suggesting another important aspect of the door cue on learning behavior. There was no difference between the Door and No Door conditions (Fig. 4*e*,*f*).

**Figure 5.**
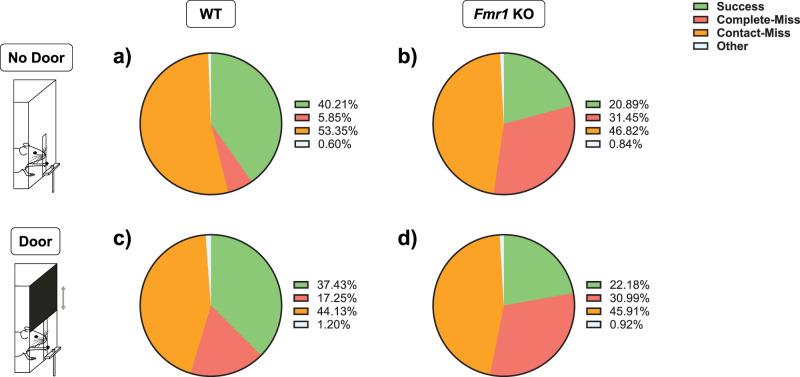
Distribution of behavioral outcomes varied between *Fmr1* KO and WT mice on the final day of learning (Day 8). ***a–d***, Successes (green), complete misses (red), contact misses (orange), and “other” (light blue) categories of reach, between both genotypes and door conditions.

In order to provide an overview of the different types of reaches we analyzed, we plotted the different reach types at the end of learning as pie charts. The categories of success, complete miss, and contact miss form the majority of reaches. The differences between the varied aspects of learning described above are apparent (Fig. 5*a*–*d*), where WT mice achieved many more successes than *Fmr1* KO mice under both conditions (WT No Door: *n* = 18, *Fmr1* KO No Door: *n* = 20, WT Door: *n* = 18, *Fmr1* KO Door: *n* = 11). However, the distribution of types of failure varied depending on the door condition.

**Figure 6.**
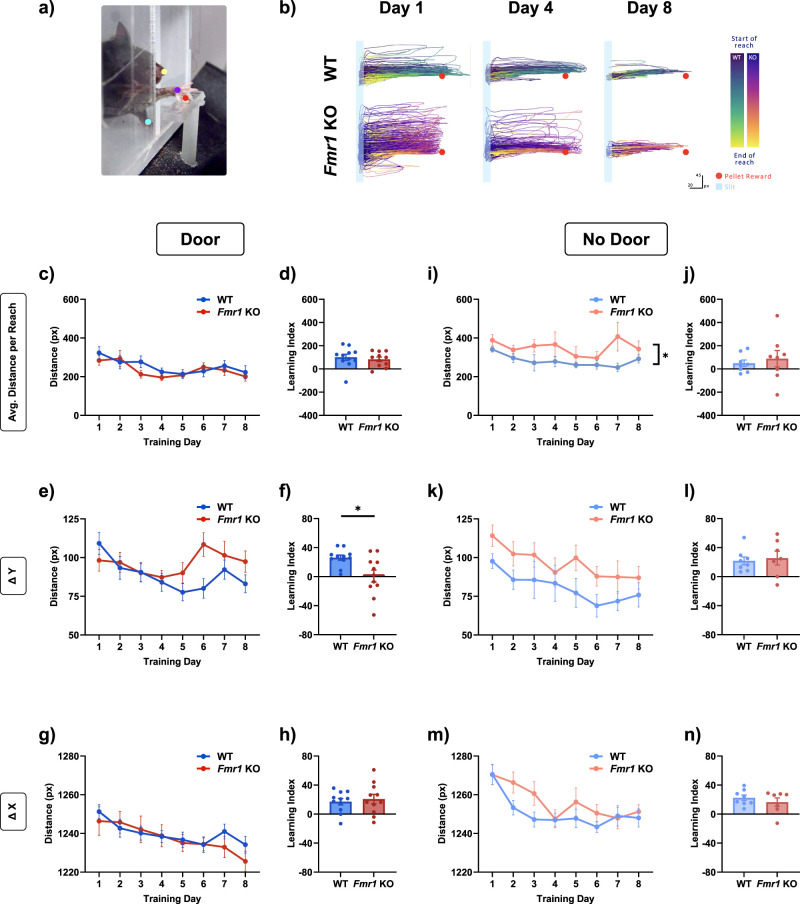
*Fmr1* KO mice showed improvement in reach trajectory over learning, but deficits in learning. ***a***, Example movie frame with the paws, pellet, and nose labeled with our DeepLabCut model. See also Movie 1. ***b***, Cumulative traces of reach trajectories over learning for individual example mice of each genotype. The reach is tracked from the slit at the left toward the target pellet reward, marked as the red dot, on the right. For visual clarity, trajectories are displayed using spline interpolation; however, all quantitative and statistical analyses were performed on raw, unsmoothed coordinates to ensure data integrity. ***c***, The time course of average distance per reach was not different between *Fmr1* KO mice and WT mice, and (***d***) neither was the Learning Index related to this metric. ***i, j***, Average distance per reach of *Fmr1* KO mice was longer than that of WT overall in the No Door condition, but there was no difference in the Learning Index. ***e***, There was a reduction in the Δ*Y* distance over learning in WT, measured as the difference between the maximum and minimum *y*-coordinate of each reach, with a significant interaction effect between genotype and training day. This difference was also reflected in a significantly lower Learning index in *Fmr1* KO mice. ***k, l***, Δ*Y* was reduced over learning days in the No Door condition, but there was no significant difference between genotypes, nor in the Learning Index for Δ*Y*. ***g, h, m, n***, Although there was improvement overall across training in both genotypes in the Δ*X*, the maximum extent of the reach in the *x* dimension, there was no difference between *Fmr1* KO and WT mice in both conditions or in the Learning Index. Time courses (***c, e, g, i, k, m***) were compared with a two-way ANOVA with repeated-measures or with a mixed-effects model. Unpaired comparisons (***d, f, h, j, l, n***) were performed with a Welch's *t* test. The main effect of genotype is indicated in the line plots by asterisks. *p < 0.05. Details of statistical tests are described in the Results section and summarized in Extended Data Figure 1-1. See also Extended Data Figure 6-1.

Since our results above suggested that there was a difference in the reach trajectory between *Fmr1* KO and WT mice, we went on to measure these trajectories using a well-established, markerless, deep learning-based method for pose estimation, DeepLabCut (Mathis et al., 2018; Fig. 6*a*, Movie 1). Over the 8 d of training, in the Door condition, the reach trajectory was strikingly refined, in both WT (*n* = 11) and *Fmr1* KO mice (*n* = 11; Fig. 6*b*). We quantified this refinement in several ways. First, we measured the distance of each reach in pixels (Fig. 6*c*). In order to better understand the process of refinement, we also assessed each animal's overall improvement over the training period by comparing its initial and final performance; we defined this as the Learning Index, calculated by taking the difference in a given metric of a mouse's performance between Day 1 and Day 8, thereby normalizing learning to the individual animal's own baseline. Although both WT and *Fmr1* KO mice showed a reduction in their average distance per reach, there was no difference between them in the Door condition [Fig. 6*c*,*d*; two-way repeated-measures ANOVA: main effect of genotype, *F*_(1,20)_ = 0.3549, *p* = 0.558; no interaction effect between genotype and training day, *F*_(4.030,80.60)_ = 1.323, *p* = 0.2684; Welch's *t* test (Learning Index): *t*_(18.07)_ = 0.5433, *p* = 0.5936]. Next, we compared the *x* and *y* dimensions of the reach, in pixels. We noticed that *Fmr1* KO mice made reaches that were more spread out in the *y*-axis; i.e., they were suboptimal and further away from the direct path to the target. We quantified this as Δ*Y*, which was defined as the difference between the maximum *y*-coordinate and the minimum *y*-coordinate of each reach. As expected, Δ*Y* was also reduced over learning for WT mice; however, this was different for *Fmr1* KO mice: Although there was no significant genotype main effect overall between WT and *Fmr1* KO mice across training days, there was a significant interaction effect (Fig. 6*e*; two-way repeated-measures ANOVA: main effect of genotype, *F*_(1,20)_ = 0.9119, *p* = 0.351; interaction effect between genotype and training day, *F*_(4.167,83.34)_ = 4.729, *p* = 0.0015). In addition, the Learning Index was significantly different between WT and *Fmr1* KO [Fig. 6*f*; Welch's *t* test (Learning Index): *t*_(13.70)_ = 2.811, *p* = 0.0141]. This suggests that *Fmr1* KO mice were less able to refine their trajectories to reach the target efficiently, an important aspect of learning to optimize their reaching movements. We also measured a metric we defined as Δ*X*, the horizontal displacement of the paw during a reach, equivalent to the maximum reach endpoint in the *x*-axis. Reach endpoint is a key feature of skilled reaching that reflects how precisely the movement is controlled, as shown in previous work, and differences in endpoint position can reveal impairments in motor refinement (Nica et al., 2018; Becker et al., 2020). The Δ*X* of reaches provides important insight into how far the paw extends beyond the slit and whether the reach trajectory overshoots or undershoots the target. As learning progressed, reaches in both *Fmr1* KO and WT mice became overall progressively shorter and no longer overshot the target; this is reflected in the reduction of Δ*X* values; however, there was no significant difference between genotypes [Fig. 6*g*,*h*; two-way repeated-measures ANOVA: main effect of genotype, *F*_(1,20)_ = 0.1421, *p* = 0.7102; no interaction effect between genotype and training day, *F*_(3.235,64.70)_ = 1.370, *p* = 0.2587; Welch's *t* test (Learning Index): *t*_(17.65)_ = 0.4744, *p* = 0.641]. Together, these findings indicate that although *Fmr1* KO mice were able to refine their reaches over learning, they nevertheless showed impairments in the extent and optimization of their reaching movements.

10.1523/ENEURO.0126-25.2026.f6-1 Figure 6-1 **Comparison of Average Distance per Reach between the Door and No Door conditions in WT and *Fmr1* KO mice.** a) WT mice had similar reach distances in both conditions, while b) *Fmr1* KO mice had significantly shorter reaches overall in the Door condition compared to No Door. Comparisons were performed using a two-way ANOVA with repeated measures, or with a mixed-effects model. Main effect of genotype is indicated in the line plot by asterisks. ** *p* < 0.01. Details of statistical tests are described in the Results section and summarized in Extended Data Fig. 1-1. Download Figure 6-1, TIF file.

**Movie 1.** A mouse reaching during a learning trial with DeepLabCut tracking. Example video of a WT mouse making a successful reach attempt during the forelimb reach task in the Door condition, with tracking dots overlaid from DeepLabCut analysis (red, pellet; purple, right paw; teal, left paw; yellow, nose). Playback speed is 0.25×. [View online]

Our manual reach scoring analysis revealed a clear deficit in *Fmr1* KO mice, which appeared to be alleviated by the addition of the door. We sought to examine whether the presence of the door also influenced the reach trajectories of the mice and whether trajectory differences may be more pronounced in the No Door condition compared with our findings in the Door condition. Trials were conducted with the same structured timing intervals for pellet placement and removal as in the automated door setup. Consistent with our manual reach scoring analysis, *Fmr1* KO mice (*n* = 8) exhibited significantly longer reaches than WT mice (*n* = 8) in the No Door condition, indicating reduced optimization of their reach trajectories (Fig. 6*i*; mixed-effects model: main effect of genotype, *F*_(1,14)_ = 4.651, *p* = 0.0489; no interaction effect between genotype and training day, *F*_(2.668,36.59)_ = 0.8262, *p* = 0.4759). Furthermore, within the *Fmr1* KO group, reach distances were significantly longer in the No Door condition compared with the Door condition (Extended Data Fig. 6-1a; mixed-effects model: main effect of door, *F*_(1,17)_ = 12.59, *p* = 0.0025; no interaction effect between door and training day, *F*_(2.895,48.40)_ = 1.658, *p* = 0.1899), suggesting that the presence of the door had a corrective effect. In contrast, WT mice showed no significant differences between conditions, underscoring that the effect of the door on refining the distance of reach trajectories was specific to the *Fmr1* KO mice (Extended Data Fig. 6-1*b*; two-way repeated-measures ANOVA: main effect of door, *F*_(1,17)_ = 0.8192, *p* = 0.3781; no interaction effect between door and training day, *F*_(3.896,66.24)_ = 1.037, *p* = 0.3936). However, there was no significant difference in the Learning Index of reach distance between genotypes (Fig. 6*j*; Welch's *t* test: *t*_(9.221)_ = 0.5235, *p* = 0.613).

We also assessed Δ*Y* and Δ*X* in the No Door condition and found reductions across training days in both WT and *Fmr1* KO mice. However, there were no significant differences between the genotypes in either metric or their corresponding Learning Indices [Fig. 6*k*–*n*; mixed-effects model (No Door, Δ*Y*): main effect of genotype, *F*_(1,14)_ = 3.762, *p* = 0.0728; no interaction effect between genotype and training day, *F*_(3.680,50.47)_ = 0.2661, *p* = 0.8852; Welch's *t* test (No Door, Δ*Y* LI): *t*_(9.598)_ = 0.3416, *p* = 0.074; mixed-effects model (No Door, Δ*X*): main effect of genotype, *F*_(1,14)_ = 1.343, *p* = 0.266; no interaction effect between genotype and training day, *F*_(2.981,40.88)_ = 1.335, *p* = 0.2762; Welch's *t* test (No Door, Δ*X* LI): *t*_(10.48)_ = 0.8608, *p* = 0.4086].

## Discussion

Human patients with FXS demonstrate a range of motor impairments (Caravella and Roberts, 2017; Reisinger et al., 2020; Chen et al., 2022). Motor learning is ideal for investigation in animal models, in comparison with more complex human behaviors that are difficult to replicate in mice. In addition, motor learning can be precisely measured and quantified, facilitating the ability to directly link specific motor deficits to their underlying cellular or circuit mechanisms. Therefore, in order to determine motor learning deficits in the *Fmr1* KO mouse, we compared a goal-directed reach learning task between *Fmr1* KO and WT mice.

We showed that *Fmr1* KO mice were capable of a form of goal-directed forelimb reach learning in which mice had to learn to retrieve a food pellet reward through a narrow slit. In alignment with previous results, we found an impairment in this forelimb reach learning in *Fmr1* KO mice, when compared with WT mice (Padmashri et al., 2013). This impairment was previously quantified as an overall success rate. A lack of success could be the result of a variety of reasons, such as problems with reach endpoint or approach, an inability to grasp the reward pellet, or problems with retrieval. Although precise reaching analysis has been done in WT rats and mice (Azim et al., 2014; Bova et al., 2021), this has not yet been done in *Fmr1* KO mice. Intriguingly, we discovered that the addition of an automated door that signaled the start and end of the trial, and thereby provided a cue as to when the reward was available, alleviated some specific aspects of the deficit in *Fmr1* KO mice. In particular, when successes were quantified as a percentage of the total trials, there was no longer a significant difference between *Fmr1* KO and WT mice. However, a more detailed analysis did indicate that there was still an impairment in motor learning. It is possible that the behavioral changes due to a door may have to do with attention deficits, with the door providing a cue signaling the availability of the reward. This may be particularly relevant to FXS, as attention deficits have been documented in human males with the disorder (Scerif et al., 2012; Cornish et al., 2013; Schmitt et al., 2019). Evidence suggests that while males with FXS can maintain attention for brief periods of time, they struggle with sustaining attention over longer periods (Sullivan et al., 2007). However, when we observed whether the mouse was close to and facing the slit in the first few seconds of the trial as a readout of attention, we were not able to detect any differences between *Fmr1* KO and WT mice. Nor did we see any obvious improvement over learning. As described in the Results section, it is also possible that the physical presence of the door constrained early reaching attempts while it was opening, thereby preventing mistargeted reaches in the space above the reward pellet. Taken together, our data highlight that the parameters of task design are critical for fully characterizing motor deficits in *Fmr1* KO mice and that differences in experimental conditions can substantially influence the observed outcomes.

Learning in goal-directed reach tasks is often summarized by a single success score. However, this single measure reflects the combined influence of multiple parameters of learning that could contribute to an overall reduction in success rate. In order to identify the specific features underlying impaired performance in *Fmr1* KO mice, we conducted a more granular analysis of reach outcomes. First, since a mouse can make several reaches within a trial, we analyzed how success varied when scored as a percentage of trials or as a percentage of targeted reaches. Our results showed that the outcomes of these two different analyses were different, which was also apparent when comparing the Door and No Door conditions. Next, out of the multiple reach attempts a mouse could make within a trial, our analysis showed that *Fmr1* KO mice were less successful at the first reach within a trial. Furthermore, the pattern of failures they made, i.e., entirely missing the target, or making contact but failing, provides insight into different aspects of the motor deficit. Future experiments will be required to further clarify the distinctions between deficits in reach trajectories, reach kinematics, or grasping and retrieval. We also determined that, at least under the specific conditions of this task, *Fmr1* KO mice did not make a higher percentage of “vain reaches” than WT, which might have been expected given the prevalence of repetitive behaviors in ASD.

We elucidated reach-type differences more precisely by employing a markerless tracking method, DeepLabCut, and showed that reach trajectories were overall refined over the time course of learning in both WT and *Fmr1* KO mice, reflected by a reduction in the average distance of each reach. In alignment with the learning deficit observed in our manual reach analysis, we found the door cue had a significant effect on the reach trajectories of the *Fmr1* KO mice, where KO mice had shorter reaches in the Door condition than in the No Door condition, and WT mice performed as well in both conditions. This also aligns with our manual scoring data that showed *Fmr1* KO mice made a higher percentage of complete-miss reaches than WT mice. Other metrics, such as the vertical extent, showed no significant difference. However, *Fmr1* KO mice made reaches that were not as optimally directed to the target, reflected by a greater vertical extent of each reach, measured as Δ*Y*. These results suggest that *Fmr1* KO mice were not able to learn optimally targeted reaches. It is possible that the deficit shown in our manual analysis can be further explained by fine motor deficits of the digits while they grasp the pellet, which were not analyzed here as we tracked only the paw dorsum during reaches. However, additional analysis into digit-scale kinematics could reveal further insights into movement learning across different levels of motor precision. It has been found that gross and fine motor components of dexterous skill are learned over different timescales; refinement of fine digit control takes longer to refine than gross trajectories (Bova et al., 2021). Individuals with FXS exhibit both gross and fine motor impairments (Will et al., 2019), but detailed analysis of how fine motor learning evolves during skill acquisition in *Fmr1* KO mice has yet to be described. Our current study also has the caveat that our field of view was limited by the camera angle and resolution, which limited our ability to simultaneously perform precise analysis of the grasping movement of the digits. Moreover, the 2D version of DeepLabCut that was used is inherently limited by the 2D plane parallel to the camera (Mathis et al., 2018); by extending our analysis to 3D DeepLabCut (Nath et al., 2019), we can obtain high-resolution recordings suitable for digit tracking, making this an important next step. Deficits in grasping may be able to explain the similarities in some reach trajectory metrics observed between WT and *Fmr1* KO mice, despite showing a deficit in reach success rates. Therefore, a more detailed analysis may highlight aspects of the reach that we were not able to measure with our current setup. Nevertheless, even with these limitations, we were able to determine how the trajectory improved over time, and how this improvement was impaired in *Fmr1* KO mice, in both conditions.

The relevant brain regions, underlying molecular mechanisms, and potential therapeutic targets in fragile X syndrome have long been a focus of intense interest (Bear et al., 2004; Bagni and Greenough, 2005; Bagni et al., 2012; Santoro et al., 2012; Bagni and Oostra, 2013; Darnell and Klann, 2013; Contractor et al., 2015; Banerjee et al., 2018; Bagni and Zukin, 2019). Various forms of behavior and learning have also been previously studied in rodent models of FXS (Fisch et al., 1999; Baker et al., 2010; Padmashri et al., 2013; Saxena et al., 2018; Gibson et al., 2023; Schmitt et al., 2023) There is a diversity of behavioral impairments in fragile X syndrome, ranging from attention deficit hyperactivity disorder to intellectual disability and to difficulties with social behavior. However, complex cognitive tasks are difficult to model accurately in mice without some degree of anthropomorphism. In addition, linking such complex behaviors to their underlying cellular or circuit-level substrates remains challenging. In contrast, motor learning offers an ideal system that is both highly relevant to mice and that allows the ability to precisely measure and quantify it. Such precise analysis provides a basis for linking behavioral features to cellular targets, particularly in the cerebellum and the motor cortex (Padmashri et al., 2013; Becker and Person, 2019). Accordingly, our investigation of motor learning deficits in *Fmr1* KO mice establishes a framework for more detailed studies of the underlying intracellular, cellular, and circuit mechanisms of fragile X syndrome. Overall, our description of deficits in forelimb reach learning in *Fmr1* KO mice brings us closer to being able to link specific features of behavioral dysfunction to their neural correlates.

## Synthesis

Reviewing Editor: Mihaela Iordanova, Concordia University - Loyola Campus Decisions are customarily a result of the Reviewing Editor and the peer reviewers coming together and discussing their recommendations until a consensus is reached. When revisions are invited, a fact-based synthesis statement explaining their decision and outlining what is needed to prepare a revision will be listed below. The following reviewer(s) agreed to reveal their identity: Richard Courtemanche, John Green.

Overall the reviewers and editor felt the manuscript provides an important contribution to the field of motor learning. The detailed reach analysis in the WT and Fmr1 knockout mice was commendable. The critical concerns that need to be addressed are detailed below:

INTRODUCTION:

The manuscript lacks comparison with the extensive literature on reaching, which includes studies in humans, non-human primates, cats, and rodents. Key themes-particularly in task design and neurological functions-should be comparatively addressed. In the Discussion, additional rodent reaching studies should be cited and evaluated in terms of performance metrics. While Becker &Person (2019) and Padmashri et al. (2013) are mentioned, a dedicated comparative section is warranted. Foundational work by Kolb and Whishaw, as well as studies on pathological models (e.g., stroke), should be included. Padmashri et al. (2013) used a similar task and is cited, but further work by this group-along with related studies (e.g., Reiner &Dunaevsky, 2015; Suresh &Dunaevsky, 2023)-should be incorporated into the Introduction (e.g., lines 74-75) and/or Discussion (e.g., lines 424-425). Some examples include:

Whishaw IQ &Kolb B (2005): Prehension. In: The Behavior of the Laboratory Rat. New York, Oxford University Press, pp. 162-170.

Ryait H, et al. (2019): Data-driven analyses of motor impairments in animal models of neurological disorders. PLoS Biol 17(11): e3000516.

METHODS:

Additional details need to be provides in the methods:

1) The # of mice within each group (WT and Fmr1 KO) which followed the initial protocol (no door) and the one with the automated door (mentioned on line 123) should be provided in the text.

2) The data processing section in the Materials and Methods needs more information:

a. On line 164, data filtering is identified (mention of being done in Python), but not detailed. Was there any formal filtering of the kinematics? What does it mean to have a p-value for the filtering? The iterative process for determining the direction of the movement vector is described, but was this p-cutoff related to the elimination of "non reaching movements" (trajectories outside the path)? It would help if this section would provide additional details on the trajectory description, both in the processing of the kinematics, and in the "extraction" of a reach from all the potential movements.

b. Details that would also be important concern the description of the learning index that is introduced on line 327. Only given in the Results, it is described a bit too lightly.

3) Please include a statement regarding Code Accessibility.

RESULTS:

1) The statistical analyses needed additional detail and transparency. Reporting only the p-values was considered inadequate and thus more thorough statistical reporting is needed including a test value (e.g., F values), degrees of freedom, confidence intervals. Effects sizes etc Where ANOVAs were conducted, interaction effects should be reported. It is not clear what the test of normality was, and then which statistical tests used the parametric vs. non-parametric test version

2) Including subheadings by dependent measure, corresponding to the figures (e.g., success by trial; success by reach; etc) would make the results easier to follow.

3) This rating system used by the scorers should be described in more detail, particularly in the Materials and Methods section (lines 218-221).

DISCUSSION

The Discussion section on the added value of the automated door is too much in abstract. While it suggests the door serves as a cue by preventing attention drift, the authors should distinguish between the animal's body position relative to the reaching window and the absence of reaching movements. Position within the enclosure could indicate attentional engagement. This possibility could be explored using a subset of data, and the role of posture and positioning in engagement delays and task performance should be discussed further.

Minor revisions.

Line 58: suggest "Pavlovian" rather than "reflexive" since eyeblink conditioning is a form of Pavlovian conditioning.

• Line 121 introduces the 'No Door' condition before its formal section; the text should be restructured for clarity. The rationale for including the door should be presented in the Materials and Methods, ideally in the 'Forelimb reaching task with automated door' subsection. Relevant details from the Results (e.g., lines 190-193) could be moved there, and this subsection should reference Fig. 1 for visual context.

• The Methods section on DeepLabCut (lines 154-162) could provide more details on the accuracy of the tracking, for various markers, and the # of frames and trials that were correctly labeled with this algorithm.

• For Fig. 1, we can see the progression of the performance with time, the time to reach a particular level of performance (e.g., 70 or 80 %) could also be considered as a variable.

• The words "learning trajectories" are used at line 251; this terminology could lend to confusion in the context of learning during reaching performance. A different terminology would be helpful to describe change with time: something like learning "progression", or "profile", or "slope" could be considered.

• Line 268, the word "reward" could be replaced with "pellet" for specificity and clarity regarding the item reached for..

• The measurement of trajectories should be transferred from pixels to mm, with a scale provided in Fig. 6. This simple conversion will provide a greater exportability and generalization of their results across studies.

• In Fig. 6A, the door and the "slit" should be indicated. Also, the color code being used is interesting, but a bit misleading. It essentially describes the progression along the length of the path, which is not that useful. The color code could be used to provide more aggregate-based measures, such as extent or variability of the starting point, or trajectory variability.

• The reference of Padmashri et al. (2013) is incomplete (lines 501-503).

• The word "demonstrate" is used a lot in the presentation of the Results and Discussion. The use of this word should be modulated a bit - it means overall to show clearly, to prove or illustrate with many examples (Merriam-Webster dictionary) - and in some cases where it is used, this is accurate, in other cases, much less. In general, people can demonstrate (prove, provide many examples), not a single figure or result.

## Author Response

Response to Reviews Synthesis of Reviews:

Computational Neuroscience Model Code Accessibility Comments for Author (Required):

A statement regarding accessibility of the code used to analyse the behavioural data has been requested in the review synthesis.

A code accessibility statement has been added. Synthesis Statement for Author (Required):

Overall the reviewers and editor felt the manuscript provides an important contribution to the field of motor learning. The detailed reach analysis in the WT and Fmr1 knockout mice was commendable. The critical concerns that need to be addressed are detailed below:

We thank the reviewers for their assessment that our manuscript provides an important contribution to the field of motor learning. Below, we have addressed all concerns that were raised. In addition, we have gone beyond the revisions requested and added a substantial amount of new data that describes reach trajectories in the "No Door" condition. The absence of this data had led to us speculating in the text; this additional dataset now complements the reach trajectory analysis in the "Door" condition and permits clearer interpretation.

INTRODUCTION:

The manuscript lacks comparison with the extensive literature on reaching, which includes studies in humans, non-human primates, cats, and rodents. Key themes-particularly in task design and neurological functions-should be comparatively addressed. In the Discussion, additional rodent reaching studies should be cited and evaluated in terms of performance metrics. While Becker &Person (2019) and Padmashri et al. (2013) are mentioned, a dedicated comparative section is warranted. Foundational work by Kolb and Whishaw, as well as studies on pathological models (e.g., stroke), should be included. Padmashri et al. (2013) used a similar task and is cited, but further work by this group-along with related studies (e.g., Reiner &Dunaevsky, 2015; Suresh &Dunaevsky, 2023)-should be incorporated into the Introduction (e.g., lines 74-75) and/or Discussion (e.g., lines 424-425). Some examples include:

Whishaw IQ &Kolb B (2005): Prehension. In: The Behavior of the Laboratory Rat. New York, Oxford University Press, pp. 162-170.

Ryait H, et al. (2019): Data-driven analyses of motor impairments in animal models of neurological disorders. PLoS Biol 17(11): e3000516.

In response to the reviewers' feedback, we have substantially expanded the Introduction section to include a broader comparative perspective across species (humans, non-human primates, cats, and rodents) and task designs. We have incorporated the suggested citations along with additional references to discuss foundational work in forelimb movement and current work in rodents that provide a more comprehensive framework and strengthens the manuscript's integration with existing literature on reaching behavior. We have also referenced studies on pathological models. Additional rodent reaching studies have also been cited and evaluated in terms of performance metrics; however, this was done in the Introduction section rather than the Discussion section since our comparative discussion is placed in the Introduction section to provide context before the Results section.

METHODS:

Additional details need to be provides in the methods:

1) The # of mice within each group (WT and Fmr1 KO) which followed the initial protocol (no door) and the one with the automated door (mentioned on line 123) should be provided in the text.

We apologize for this omission. The number of mice from each genotype (WT and Fmr1 KO) used for each experimental condition (No Door and Door) has now been added to the text. In addition, we have specified the number of mice for each experiment in the Results section, including the breakdown by sex within each genotype.

2) The data processing section in the Materials and Methods needs more information: a. On line 164, data filtering is identified (mention of being done in Python), but not detailed. Was there any formal filtering of the kinematics? What does it mean to have a p-value for the filtering? The iterative process for determining the direction of the movement vector is described, but was this p-cutoff related to the elimination of "non reaching movements" (trajectories outside the path)? It would help if this section would provide additional details on the trajectory description, both in the processing of the kinematics, and in the "extraction" of a reach from all the potential movements.

The "p-cutoff" that is used for movement analysis is a probability value described by Mathis et al., 2018 in the DeepLabCut process: all the tracked datapoints outputted from DLC yields corresponding "p-cutoff" values, which assigns each datapoint with a likelihood value of how confident the model is that the tracked datapoint in a given frame is accurate. Filtering was performed on a point-by-point basis, such that only datapoints exceeding a defined confidence threshold were retained for subsequent kinematic and trajectory analyses. Once the positions of the labeled body parts in each frame were filtered, we then filtered all the data again on a point-by-point basis to ensure physical plausibility, removing any confidently marked points that were outside of the bounds of the possible reaching space. This eliminated any possible errors in labeling and noise from other non-reaching related behaviors inside the box that could affect the reaching analysis. Reach extractions were then performed on this filtered data. We have now provided additional details in the Methods section (lines 239-260) to clarify the definition and use of the "p-cutoff" value. b. Details that would also be important concern the description of the learning index that is introduced on line 327. Only given in the Results, it is described a bit too lightly.

The Learning Index quantifies each individual animal's overall improvement across the training period by comparing its initial and final performance, thereby normalizing learning to the individual animal's own baseline. We have expanded the description of the Learning Index (lines 508-510) to clarify its purpose and calculation, which should provide clearer context for interpreting the Learning Index in relation to the other performance measures discussed.

3) Please include a statement regarding Code Accessibility.

A Code Accessibility statement has now been added to the Methods section (lines 265-272). RESULTS:

1) The statistical analyses needed additional detail and transparency. Reporting only the p-values was considered inadequate and thus more thorough statistical reporting is needed including a test value (e.g., F values), degrees of freedom, confidence intervals. Effects sizes etc Where ANOVAs were conducted, interaction effects should be reported. It is not clear what the test of normality was, and then which statistical tests used the parametric vs. non-parametric test version We apologize for the lack of clarity regarding statistical tests and thank the reviewers for this constructive feedback. We have substantially expanded our statistical analysis reporting throughout the Results section and have added more details to our Statistical Methods section to describe all statistical tests performed. All test statistics (e.g. F values), degrees of freedom, effect sizes, confidence intervals, and significance values are now compiled into a table in Table 2 and summarized in the Results section.

2) Including subheadings by dependent measure, corresponding to the figures (e.g., success by trial; success by reach; etc) would make the results easier to follow.

We agree that adding subheadings improves flow. Subheadings corresponding to the dependent measures and figures have been added throughout the Results section to improve clarity and readability.

3) This rating system used by the scorers should be described in more detail, particularly in the Materials and Methods section (lines 218-221).

More details regarding the reach scoring system, including the specific criteria used by the scorers, have been added to the Methods section. We have added more detail to the reach classification summary table (Table 1) for clarity of the different reach types, and have referenced this table with a brief description again in the text (lines 197-210).

DISCUSSION The Discussion section on the added value of the automated door is too much in abstract. While it suggests the door serves as a cue by preventing attention drift, the authors should distinguish between the animal's body position relative to the reaching window and the absence of reaching movements. Position within the enclosure could indicate attentional engagement. This possibility could be explored using a subset of data, and the role of posture and positioning in engagement delays and task performance should be discussed further.

We agree with the reviewers that in the previous version, our discussion on the added value of the automated door was too abstract. The major reason for this was that we did not have reach trajectory analysis in DeepLabCut for reaches performed in the No Door condition, and thus could only speculate as to whether the reach itself was different due to the Door and what the reasons for that might be. We now performed new experiments and have added new data with mice in the No Door condition, which include DeepLabCut reach trajectory tracking (Figure 6). Therefore, we have significantly improved the interpretability of the Door vs. No Door experiments.

Regarding the possibility of differences in attentional engagement, which we had earlier suggested when we did not have the No Door reach trajectory data, we addressed the concern above by investigating body position, as suggested. We had speculated that when the door cue was absent, mice that were not paying attention were more likely to miss the start period of the trial, thereby having less time to make reach attempts before the pellet reward was removed. Therefore, we quantified each mouse's attentiveness during each trial to see if attention improved over learning, or was different in Fmr1 KO mice, by tracking their nose. We presumed if the nose was within the vicintiy of the slit, the mouse must be facing the slit and therefore was attentive of the pellet reward. For each trial, if the nose was within (100 px) of the slit within the first 3s of the trial, we scored the trial as "attentive", and if the nose was not present before then, the trial was marked as "inattentive". We found that there was no significant difference in attentiveness between WT and Fmr1 KO mice with these parameters (Figures below). However, the data on nose location show high variability between individuals (see error bars in figures below), so we cannot draw firm conclusions about genotype differences. We thank the reviewers for their insightful suggestion; based on this analysis, we have no basis for suggesting attention deficits or differences in posture or positioning. Therefore, we no longer make this a major focus in the text.

Minor revisions.

Line 58: suggest "Pavlovian" rather than "reflexive" since eyeblink conditioning is a form of Pavlovian conditioning.

The text has been edited to use "Pavlovian" instead of "reflexive". • Line 121 introduces the 'No Door' condition before its formal section; the text should be restructured for clarity. The rationale for including the door should be presented in the Materials and Methods, ideally in the 'Forelimb reaching task with automated door' subsection. Relevant details from the Results (e.g., lines 190-193) could be moved there, and this subsection should reference Fig. 1 for visual context.

The introduction of the No Door condition before its formal section has been removed and a new subsection called "Forelimb reaching task with an automated Door" has been created, with Figure 1 referenced. Although we still have a short description of the automated door in the Results section to aid the flow of the text, relevant details have been added to the Methods section. • The Methods section on DeepLabCut (lines 154-162) could provide more details on the accuracy of the tracking, for various markers, and the # of frames and trials that were correctly labeled with this algorithm.

We have clarified and expanded the description of the model's accuracy and performance in the Methods section (lines 244-249). While the test error is the standard metric for reporting model accuracy, we recognize the value in providing additional context on this metric and the dataset used. To provide additional clarity, we have added details specifying that the model was trained using 95% of the manually labeled frames, and the remaining 5% was set aside for testing. By definition, the test error of a deep learning model is calculated by taking a percentage of the total number of labeled inputs (frames, in this case), and the resulting accuracy metric, reported as a 3.44 pixel test error, reflects the average prediction error across all tracked points on this 5% independent testing set. We believe the additional detail added to the text now provides clarity on the model's performance. • For Fig. 1, we can see the progression of the performance with time, the time to reach a particular level of performance (e.g., 70 or 80 %) could also be considered as a variable.

We thank the reviewers for this suggestion - we explored analysis using the time (number of days) to reach a particular performance level as a potential variable; however, we found this analysis to be an unreliable measure for our data. Because individual mice exhibit daily fluctuations in success rates, a single "good day" could trigger a threshold somewhat stochastically. For example, if two mice performed similarly, and one had triggered the threshold on Day 4 by a difference of one successful trial, while the other triggered the threshold on Day 8 for the same reason, this could significantly shift the "time to success" metric, and the time to reach a level of performance or peak may just reflect the noise of behavioral performance. Therefore, we believe that analyzing the full progression of learning in the two ways we currently show it (progression over 8 days and Day 1 vs. Day 8 comparison) is a more accurate reflection of the learning process, as it accounts for the consistency of performance overall rather than relying on a single data point that may be skewed by session-to-session variability. • The words "learning trajectories" are used at line 251; this terminology could lend to confusion in the context of learning during reaching performance. A different terminology would be helpful to describe change with time: something like learning "progression", or "profile", or "slope" could be considered.

We agree with this suggestion. "Trajectories" has been updated to "progression of learning" to improve clarity. • Line 268, the word "reward" could be replaced with "pellet" for specificity and clarity regarding the item reached for.. "Reward" has been updated to "pellet reward" for clarity and specificity. • The measurement of trajectories should be transferred from pixels to mm, with a scale provided in Fig.

6. This simple conversion will provide a greater exportability and generalization of their results across studies.

We had previously considered converting our reported trajectory measurements from pixels to millimeters (mm) for the reasons mentioned by the reviewer. However, because our camera was positioned at an angle to the reach plane, the pixel to mm conversion will vary depending on the position of the forearm. Therefore, this conversion is not trivial as a straightforward pixel-to-mm conversion would not accurately reflect true spatial distances. Reporting our data in pixels allows us to reliably quantify relative differences and trends across conditions, sufficient to determine a phenotype without implying misleading spatial accuracy. We acknowledge this limitation and plan on addressing it in our future experiments using recordings from additional angles for 3D tracking, using both marker-less and optical motion capture methods. For the current study, we believe retaining pixel units provides the clearest and most accurate representation of the data given the constraints of our recording setup. • In Fig. 6A, the door and the "slit" should be indicated. Also, the color code being used is interesting, but a bit misleading. It essentially describes the progression along the length of the path, which is not that useful. The color code could be used to provide more aggregate-based measures, such as extent or variability of the starting point, or trajectory variability.

We appreciate the reviewer's suggestion and understand the interest in using the color code to convey more aggregate measures such as trajectory variability or starting point extent. We respectfully would like to clarify that the primary purpose of the trajectory traces in Fig. 6b (previously Fig. 6a) is solely to provide a representative visual example of reach trajectories to help readers understand the nature of the tracking data used in subsequent analyses. It is not intended to convey quantified results or to make claims about trajectory variability. Incorporating color to reflect variability, while interesting, could distract from this purpose and potentially mislead readers into assuming that variability is analyzed in the following figures, which it is not. That said, we have revised the figure to clearly label the slit, as suggested. The door is open at the time the camera is recording the paw's trajectory and is therefore not in this image. • The reference of Padmashri et al. (2013) is incomplete (lines 501-503). We apologize for this error and have now corrected it. • The word "demonstrate" is used a lot in the presentation of the Results and Discussion. The use of this word should be modulated a bit - it means overall to show clearly, to prove or illustrate with many examples (Merriam-Webster dictionary) - and in some cases where it is used, this is accurate, in other cases, much less. In general, people can demonstrate (prove, provide many examples), not a single figure or result.

We agree with the reviewer's comment regarding the usage of the verb "demonstrate" and the suggestion for more precise terminology. We have revised the Results and Discussion sections to use more appropriate words.
