# Supplementary figures and images for: Deficits in Forelimb Reach Learning in a Mouse Model of Fragile X Syndrome
Source: eNeuro. 2026 Apr 14;13(4):ENEURO.0126-25.2026. doi: 10.1523/ENEURO.0126-25.2026 (PMC13095381; doi:10.1523/ENEURO.0126-25.2026)

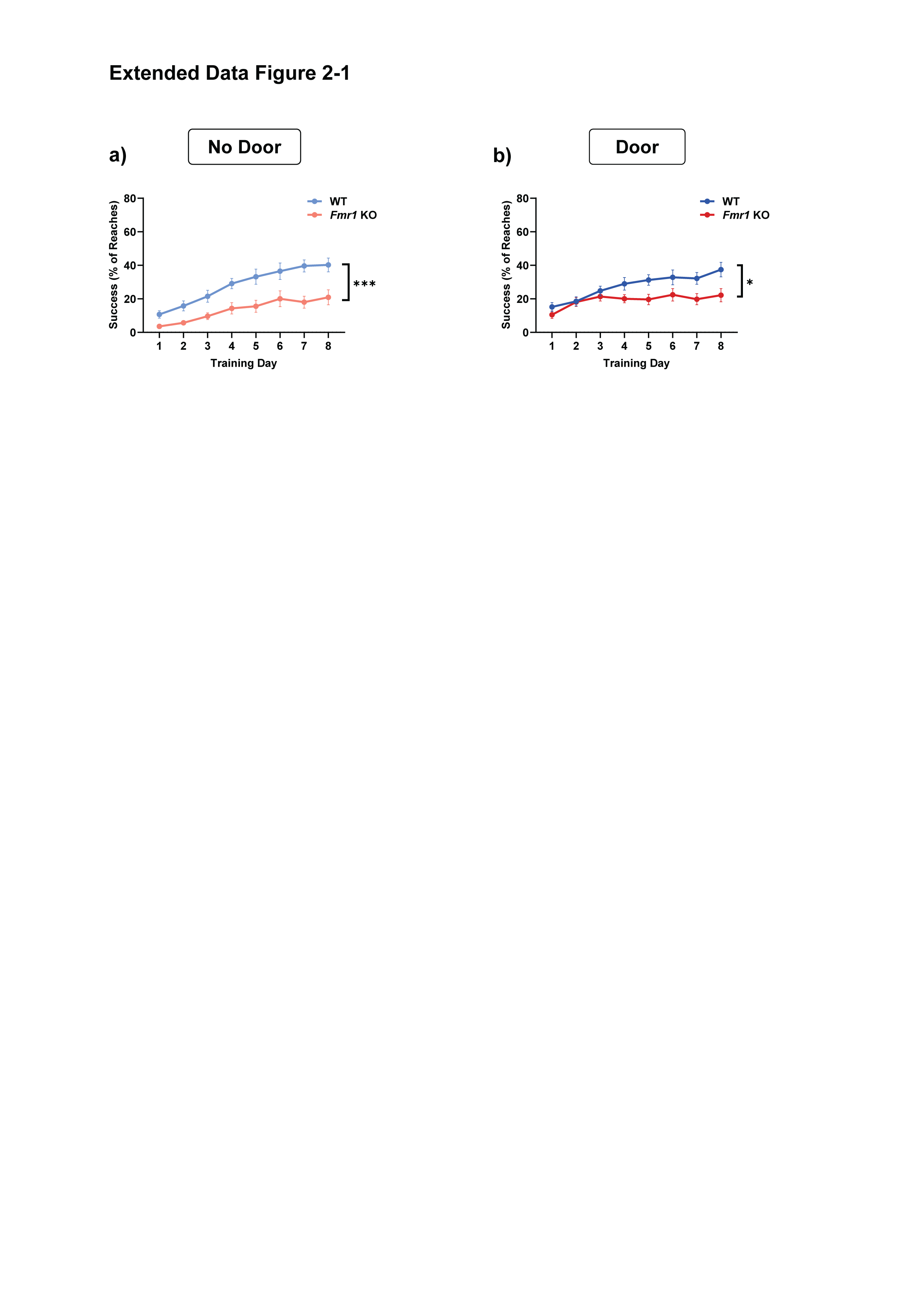

Supplement: Figure 2-1 — Validation of behavioral scoring by scorers blind to genotype. Reach analysis was performed by additional scorers who were blind to genotype, which replicated the results of Figure 2. a, b) Fmr1 KO mice showed significantly impaired learning in both the No Door and the Door condition. Comparisons were done using a two-way ANOVA with repeated measures, or with a mixed-effects model. Main effect of genotype is indicated in the line plots by asterisks. *** p = 0.001, * p < 0.05. Details of statistical tests are described in the Results section and summarized in Figure 1-1: Statistics table. Detailed statistical results table for all analyses in all figures, including test type, degrees of freedom, F-statistic (or other applicable statistic), p-values, effect sizes, and confidence intervals. Download Figure 2-1, TIF file. [file eneuro-13-ENEURO.0126-25.2026-s002.tif]

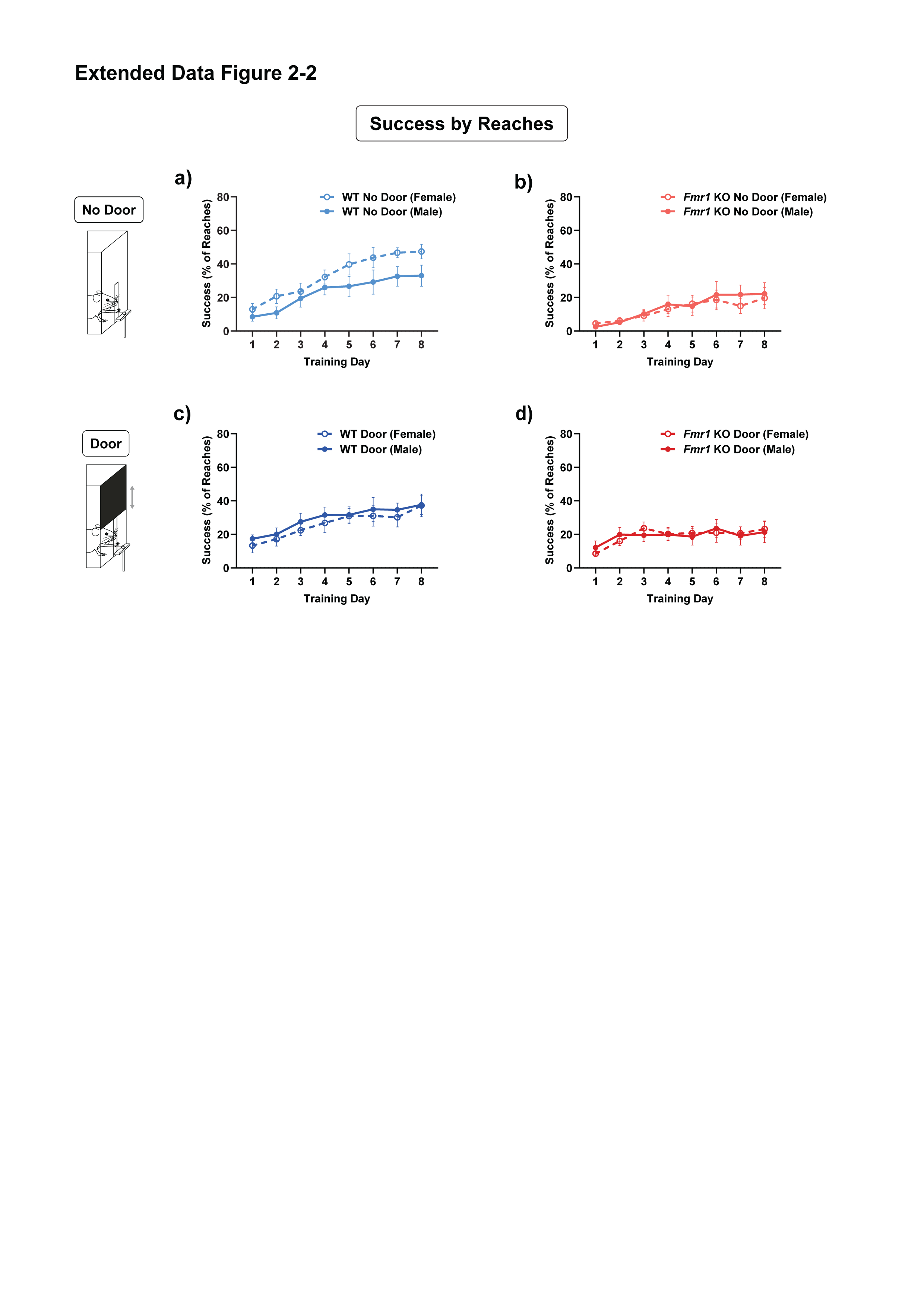

Supplement: Figure 2-2 — Comparison of sex differences in WT and Fmr1 KO mice. There was no difference in learning between sexes. a-d) There was no significant difference between male and female mice in the time course of learning for both genotypes and for both Door and No Door conditions. Comparisons were done using a two-way ANOVA with repeated measures, or with a mixed-effects model. Details of statistical tests are described in the Results section and summarized in Extended Data Fig. 1-1. Download Figure 2-2, TIF file. [file eneuro-13-ENEURO.0126-25.2026-s003.tif]

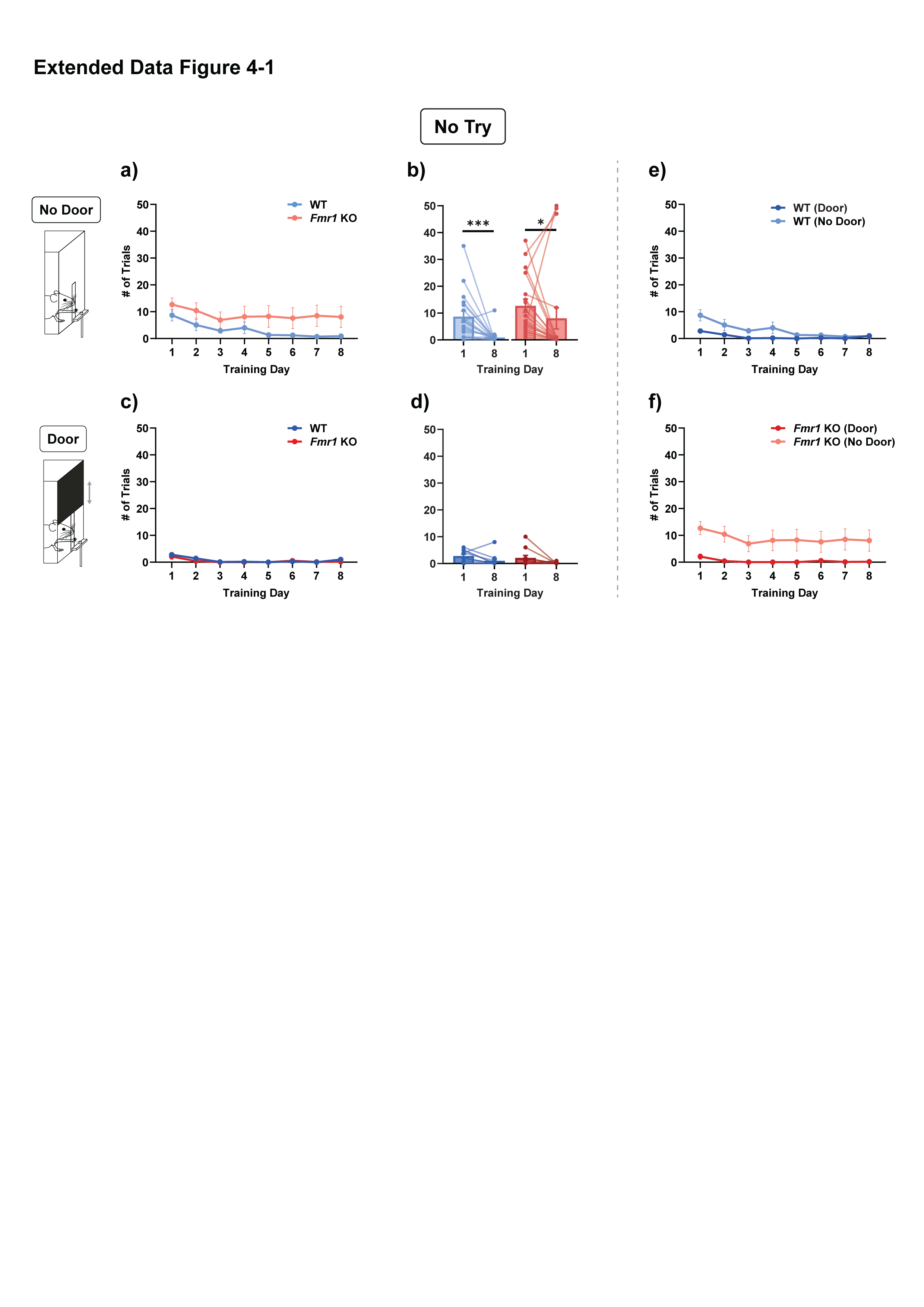

Supplement: Figure 4-1 — Comparison of “No Try” trials between WT and Fmr1 KO mice in the No Door and Door conditions. No Tries varied between the Door and No Door conditions. a, b) There was a reduction in No Tries in WT and Fmr1 KO mice in the No Door condition, indicating an improvement in performance. c, d) The number of No Tries were comparably low on both Day 1 and Day 8 for WT and Fmr1 KO mice in the Door condition. e, f) Same data as in a, c, comparing Door vs. No Door conditions between genotypes. Paired comparisons (b, d) were performed with a paired t-test or Wilcoxon matched pairs signed rank test. *** p = 0.001, * p < 0.05. Details of statistical tests are described in the Results section and summarized in Extended Data Fig. 1-1. Download Figure 4-1, TIF file. [file eneuro-13-ENEURO.0126-25.2026-s004.tif]

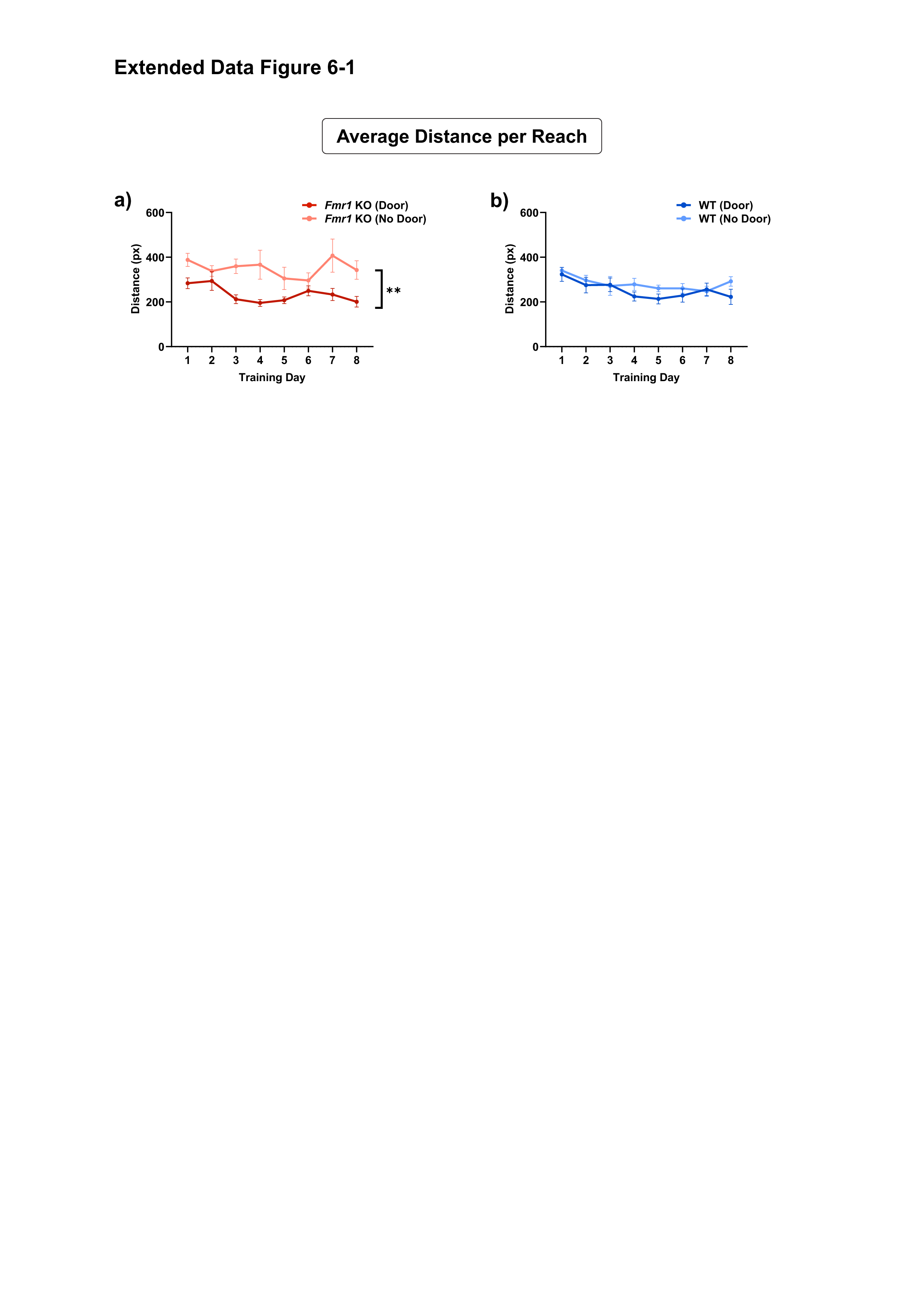

Supplement: Figure 6-1 — Comparison of Average Distance per Reach between the Door and No Door conditions in WT and Fmr1 KO mice. a) WT mice had similar reach distances in both conditions, while b) Fmr1 KO mice had significantly shorter reaches overall in the Door condition compared to No Door. Comparisons were performed using a two-way ANOVA with repeated measures, or with a mixed-effects model. Main effect of genotype is indicated in the line plot by asterisks. ** p < 0.01. Details of statistical tests are described in the Results section and summarized in Extended Data Fig. 1-1. Download Figure 6-1, TIF file. [file eneuro-13-ENEURO.0126-25.2026-s006.tif]
